# Comparative analysis of medicinal plants used in traditional medicine in Italy and Tunisia

**DOI:** 10.1186/1746-4269-5-31

**Published:** 2009-10-26

**Authors:** Maria Lucia Leporatti, Kamel Ghedira

**Affiliations:** 1 Dipartimento di Biologia Vegetale, Università "La Sapienza" Roma, Italy; 2 Laboratoire de Pharmacognosie, Faculté de Pharmacie, Rue Avicenne, 5000 Monastir, Tunisia

## Abstract

**Background:**

Italy and Tunisia (Africa for the Romans), facing each other on the opposite sides of the Mediterranean Sea, have been historically linked since the ancient times. Over the centuries both countries were mutually dominated so the vestiges and traces of a mutual influence are still present. The aim of the present study is to conduct a comparative analysis of the medicinal species present in the respective Floras in order to explore potential analogies and differences in popular phytotherapy that have come out from those reciprocal exchanges having taken place over the centuries

**Methods:**

The comparative analysis based on the respective floras of both countries takes into consideration the bulk of medicinal species mutually present in Italy and Tunisia, but it focuses on the species growing in areas which are similar in climate. The medicinal uses of these species are considered in accordance with the ethnobotanical literature.

**Results:**

A list of 153 medicinal species belonging to 60 families, present in both floras and used in traditional medicine, was drawn. A considerable convergence in therapeutic uses of many species emerged from these data.

**Conclusion:**

This comparative analysis strengthens the firm belief that ethno-botanical findings represent not only an important shared heritage, developed over the centuries, but also a considerable mass of data that should be exploited in order to provide new and useful knowledge.

## Background

**Figure 1:**
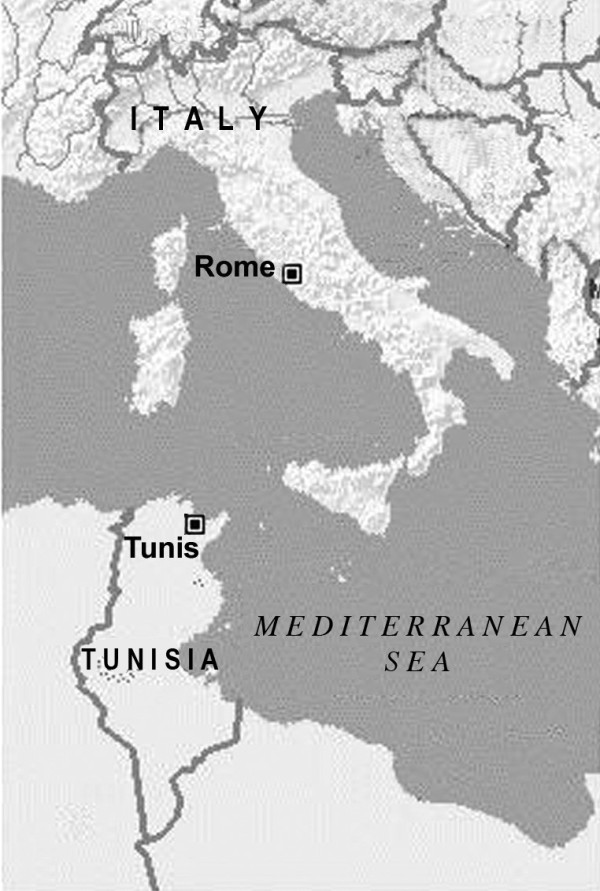
Map of investigated area

Italy and Tunisia (Africa for the Romans), facing each other on the opposite sides of Mediterranean Sea [Figure 1], have been historically linked since the ancient times and they still show the vestiges of a mutual influence. In fact both countries were themselves mutually dominated. The Roman Conquest began with the Punic wars (from 264 to 146 BC) whereas Arabian domination in southern Italy, mainly in Sicily, took place in the 9th century. The traces of both dominations were so important and long lasting that they are still present even now, not only as archaeological evidences but also as names of Italian towns or localities, e.g. Caltanissetta, Caltagirone, these toponyms derive from arabian *Kalaat *= castle; Gibilmanna, Gibellina from *gebel *= mountain, Marsala from *marsa *= port, and the town Mazara del Vallo from *Mazraet el wali *that means *Field of the Governor*. Many other examples can be given. In Tunisia, the name of the city of Ghar el Melh, till the independence of Tunisia (1956), was *Porto Farina*. Two small islands, situated in the north-east of the bay of Tunis, are named Zembra and Zembretta. The 7 km road which connects the isle of Jerba to the continent (Tunisia), built by the Romans, is still in service and named "The Roman roadway" [1].

The landscapes of these countries alternate fertile flatlands to mountainous chains.

In Tunisia, the typical Mediterranean climate which prevails in the Northern and Eastern part of the country collides with the desertic one in the South. In Italy, the climate is generally temperate; although, from North to South, three distinct climatic belts are distinguishable. People living in rural areas have been engaged in agriculture. In the Tunisian villages far from the towns, popular or traditional use of medicinal plants is still familiar to ordinary people. Whilst in Italy, it survives mainly amidst shepherds and older farmers.

## Methodology

The aim of the present study is to compare the medicinal species present in both floras as well as their therapeutic uses. Tunisian floras [2-5] consider as medicinal about 200 species among the 2150 recorded. Italian flora by Pignatti [6] lists 5559 species, among which about 1163 are considered medicinal [7]. Specific ethnobotanical literature of both countries has been consulted. The considered plants are mainly those growing in areas of northern and central Tunisia, as well as in the regions of central and southern Italy (so including Abruzzo, Marche and Toscana regions all belonging to Central Italy and where the same medicinal species are used). We excluded areas such as Apennines and Alps, or more generally northern Italy and the arid and desertic regions of central and southern Tunisia. So a list of about 153 medicinal species (belonging to 60 families), has been drawn, according to specific ethnobotanical literature [8-48]

## Results

In additional file 1 we reported the results of the present comparative study. For each species we have included the following details: scientific and vernacular name, therapeutic properties, used part, preparation and eventual additional note. The interesting therapeutic uses are highlighted in bold, as well as the mutual ones. Most of the documented plants are involved in the cure of more than one disease and this is recorded in different apparatuses. So the 153 species have a manifold of different uses exceeding more and more the number of the quoted species. Because of the high number of recipes and customs of plant uses in the different considered areas, information about their manipulation is necessarily synthetic e.g. infusion, decoction, and so on, without further specifications about doses or time required for the cure. The relative percentage of use for each single apparatus has been calculated on the basis of the total amount of uses.

## Discussion

### 1-Digestive system

One hundred and fifteen species are used to treat troubles of gastro-intestinal tract. About 23 of them have the same or comparable therapeutic uses in both countries and 15 species have identical manipulation as far as the drugs are identical. Only one species is used for really opposite purposes: *Globularia alypum*. In Italy, it is considered a purgative and in Tunisia it is a remedy for gastric ulcer [12,17,26,28,29]. It seems amazing since a purgative generally acts as a stimulator of peristalsis whilst a spasmolytic action is beneficial for gastric ulcer. It is interesting to observe that the species are considered not only as generically digestive, appetizer, aromatizing or carminative etc..., but are claimed to be effective in the case of significant ailments, e.g jaundice (e.g. *Ecballium elaterium *(L.) A. Richard, *Cichorium intybus *L., *Marrubium vulgare *L. *Rhamnus alaternus *L.) or gastric ulcer (*Pimpinella anisum *L. *Artemisia campestris *L. *Conyza canadensis *L., *Glycyrrhiza glabra *L.). Numerous species are employed for gastralgia, including the spasmolytic ones and those soothing colic pain (e.g. *Anethum graveolens *L., *Mentha spicata *L., *Malva sylvestris *L. *Polygonum aviculare *L., et).

The number of liver protecting species, such as *Cichorium intybus *L., *Cynara scolymus *L., *Silybum marianum *L., *Sonchus oleraceus *L. *Raphanus raphanistrum *L. *Marrubium vulgare *L., is also relevant. This protection is essentially due to their cholagogue, choleretic and depurative activities. Most of the species are used mainly as purgative or antidiarrhoeic remedies.

### 2- Cutaneous system

There are 127 species involved in the skin care: this is the highest number of species among all of the groups. Twenty-eight of them have similar uses, but only 4 (*Artemisia campestris *L. [9-11,21], *Borago officinalis *L. [7,9,14-18,22,24-26,28-30,45,47,48]* Capsella bursa-pastoris *L. [10,14,18,25,26,48] and *Ficus carica *L. [7,12,15-18,20,24,28,29,31,41,45,47,48]) have identical uses in both countries. The differences in uses between the two countries are remarkable. In Italy, the main frequent uses of these species are cosmetic, lenitive, emollient or cicatrizing of sores and little wounds, or at least, for minor troubles as furunculosis, chilblains, warts and corns. The uses are nevertheless interesting: *Ecballium elaterium *(L.) A. Richards, *Verbascum sinuatum *L. and *Verbena officinalis *L. were used for psoriasis treatment in Sicily [9]. These plants, from a different phylogenetic point of view and exhibiting strongly different chemical contents, are collected in spring and used in mixtures with other species not quoted in the present list. A decoction, prepared with hot water and untreated wine, is boiled and left to evaporate at room temperature then filtered and stored in a refrigerator [8].

In the Sardinian region, the so called "sapa" which is the boiled must of *Vitis vinifera *L., is considered effective for psoriasis [10].

On the contrary, in Tunisia, a considerable set of species fights serious illnesses e.g. eczema and dermatosis. Noteworthy among these species is the *Nerium oleander *L. used as antigangrenous, *Citrullus colocynthis *(L.) Schrad. and *Linum usitatissimum *L. used for impetigo, a skin infection due to the *Streptococcus *bacteria. *Ricinus communis *L. leaves are employed in soothing acne not a very serious but unpleasent illness affecting mostly young people. Moreover, sap from *Solanum nigrum *L., generally considered as toxic plant, is used for erysipelas that is due to *Staphylococcus *sp. and *Streptococcus *sp., two pyogenes bacteria. In Tunisia *Lycium europaeum *L., *Morus alba *L. and also *Solanum nigrum *L. are used for eczema.

### 3- Circulatory system

This set of 73 species diverges in the two countries and not only in the therapeutic uses. Only 9 species are used in both countries and 3 of them have the same use (*Cupressus sempervirens *L. [7,16-18,27-29], *Allium sativum *L. [7,9,17,18,29] and *Olea europaea *L. [7,13-18,20,22,28,29,39,40,42,48]) while the others clearly differ: e.g. in Italy 3 species (*Fumaria capreolata *L., *Ruta graveolens *L. and *Vitis vinifera *L.) are claimed to improve blood capillary vessel circulation. The Tunisian use of *Allium cepa* L. in case of stroke is noteworthy, whilst *Allium sativum *L. is considered as a good cardiotonic medication. Many of these species such as *Pistacia lentiscus *L., *Calendula arvensis *L., *Capparis spinosa *L. or *Lycopersicum esculentum *L. are employed for the treatment of very serious diseases such as hypertension or cardiac diseases e.g. *Carum carvi *L., *Conyza canadensis *L., *Marrubium vulgare *L., *Teucrium capitatum *L. and *Nigella damascena *L. Species such as *Cytrullus colocynthis *(L.) Schrad., *Allium sativum *L., *Marrubium vulgare *L. are employed to treat haemorrhoids or varicose ulcers.

### 4- Skeletal and muscular system

Among the 67 species used for this purpose, only 9 are used in both countries. Twenty-nine are used in Tunisia and act exclusively as antirheumatic or in painful joints. A very small number of species also have a role in soothing muscular pain (lumbago). The 25 Italian ones are also used mainly for this purpose, but in Italy some species have precise indication for gout, arthritic and neuralgic (sciatic and trigeminal) pains e.g. *Conyza canadensis *L., *Capparis spinosa *L., *Sambucus nigra *L., *Lavandula stoechas *L., *Ranunculus bulbosus *L. A small number of species give relief to muscular pains like the stiff neck e.g. *Melissa officinalis *L., *Rosmarinus officinalis *L. It is important to point out that not all species have a really anti-inflammatory role, but act as revulsive, fit for healing locally the painful part, or worse, act only as vesicatory as *Thapsia garganica *L. and *Arum italicum *Miller. These two species are able to provoke erythema or even skin damage. Only *Sambucus nigra *L. is used in case of dislocated bones (sprains), and it is effective against the swelling consequent to the dislocation [7,12,16-18,28-30].

### 5- Oral hollow, eye and ear

Looking at the repertory of 64 plants concerning troubles of mouth, eye and ear, we noted that in Tunisia people have recourse to these plants more frequently than in Italy (38 and 17 species are respectively quoted). Most part of the uses refer generally to treatment of inflammation of the mouth and throat mucous membrane etc., or also to soothe toothache (decay only in one case of pyorrhea) and gingival abscesses as mouth wash and gargling. Only *Ficus carica *L. [7,16-18,28,29], *Punica granatum *L. [7,17,18,28,45] and *Rubus ulmifolius *L. [7,17-19,28,45] are used in both countries for gingivitis treatment. Inflammation of the eye and ear are treated by eye salve and drops to instill. Two species: *Reseda alba *L. and *Vitis vinfera *L. [7,16,17,27-29] share an identical use. It is very interesting to note that several Tunisian prescriptions are used against important eye diseases e.g. *Sonchus oleraceus *L. and *Reseda alba *L. in case of ocular leukoma. Whilst *Marrubium vulgare *L. and *Nigella damascena *L. are considered effective in case of trachoma. Cattle and sheep trachoma is on the contrary treated by the fruits of *Capsicum annuum *L. Concerning ear troubles, a considerable number of species are employed in Tunisia whereas only *Olea europaea *L. is used in Italy.

### 6- Respiratory system

It is not easy to focus clearly on the role of the 72 employed species in the troubles of respiratory tract because, compared to the other systems, they have a larger fan of widely overlapping activities. So the emollient and the lenitive ones can act at the same time as antitussive, expectorant, mucolytic and hence are useful in bronchitis, laryngitis or in flu and cold. In Tunisia, the species are mainly used as antitussive, whilst in Italy they are considered also as generic expectorant, balsamic and lenitive. Only 16 species are employed in both traditional phytotherapies, but 7 of them have the same purposes. Among these, five are mutually used as antitussive. Some interesting properties must be noted. In both countries, the popular experience recognizes the effectiveness of *Raphanus sativus *L. for whooping cough [17,18,28,29]. It is remarkable that this property is completely unknown in Central and Southern Italy. On the contrary, in Veneto region and in the near north-eastern areas, people commonly have recourse to this plant.

According to the Tunisian tradition, asthma benefits from the use of several plants e.g. *Pistacia lentiscus *L., *P. terebinthus *L. and *Ajuga iva *(L.) Schreb., whilst in Italy, the properties of these species are not known. *Marrubium vulgare *L. in Tunisia and two species of *Myrtaceae *(*Eucalyptus *sp. pl. and *Myrtus communis *L.) are claimed in Italy as antiseptic for the lungs. In Italy, the importance ascribed to *Equisetum telmateja *Ehrh. as restructuring of lung cartilage must not be undervalued owing to its richness in Silicium (it is also important in the treatment of bone trouble).

### 7- Urinary system

Among the 66 species involving the urinary apparatus only 10 are used in both countries, and 9 are prescribed in both countries to promote diuresis which is the main application of the considered entities. The species used for the treatment of kidney stones differ widely, but only one of them is reported in both countries: *Ammi visnaga *(L.) [16-18,26,28,29]Lam. In Tunisia, a small number of them have antiseptic properties or are employed in children's enuresis (*Carum carvi *L. [16-18,28,29])

### 8- Antipyretic and Head ache

The 35 species of this group, related to both symptoms, are considered altogether since the headache is often a consequence of the fever or it is a simultaneous symptom. The number of species employed in Tunisia is higher than those used in Italy. In Tunisia, the popular medicine suggests in fact 11 species against headache but none of them is used in Italy for the same purpose.

Many species have a generic antipyretic employ, others are used specifically for malarial fever e.g. *Globularia alypum *L. (in Tunisia) [16-18,29], *Artemisia absinthium *L. [7,27], *Buxus sempervirens *L. [27,37,45] and *Marrubium vulgare *L. [27] (in Italy). In Tunisia, *Ammi visnaga *(L.) Lam. [16-18,28,29] and *Cucurbita pepo *L. [17,18,29] are considered in the treatment of thyphoid fever. However it is not really clear if their activity concerns only the symptom fever or have any capacity to fight the respective pathogenous agents: *Plasmodium *and *Rickettsia*. In Tunisia, *Helianthus annuus *L. and *Allium cepa *L. are used to treat fever due to sunstroke [16-18,29]

### 9- Antihelmintic, antiparasitic, etc...

The 37 species of this group, except the antihelmintic ones, have chiefly an external use in repelling insects or skin parasites. Few of them are generically considered as antiseptic and antibacterial agents without any specification about which kind of bacteria they act against. Among the species listed, four are equally considered antihelmintic.

*Cucurbita pepo *L., *Globularia alypum *L. and *Corydothymus capitatus *(L.) Reichenb. Fil. are specific in Tunisia for tinea [16-18,28,29], whilst in Italy the latter is considered effective specifically against *Ancylostoma duodenalis*, a Nemathelminthe worm [7]. These data point out the use of *Cucurbita pepo *L. and *Globularia alypum *L., respectively in the treatment of malaria and thyphoid fevers and they strenghten the assumption of a real effectiveness against several kinds of pathogenous agents.

### 10- Hypoglycemic

This little set of species (fifteen), 9 in Tunisia and 6 in Italy, is of great importance. Only one of them (*Cichorium intybus *L. [7,16-18,27-29]), is reported in both countries for the ability to lower blood level of glycemia, since diabetes is a very serious disease affecting millions of people in the world. So it is strange enough that in the two countries the plants used for this purpose are so different. In any case, their action is likely more preventive than substantially therapeutic especially when the illness is already diagnosed. The use of *Allium cepa *L. as well as *Lupinus sp. pl*. well known remedies, is strongly limited by the presence in the seeds of lupanine and lupinine alkaloids which are very dangerous to renal apparatus that can't be used without severe side effects.

### 11- Reconstituent, Vitaminic action

The 27 reconstituent species include mainly those with a large range of activity like vitaminic (chiefly A, C), tonic, remineralizing (Fe, Si) appetizer, etc. Four species are used in both countries: *Nasturtium officinale *R.Br. [7,18,26,28,29,45], *Medicago sativa *L. [18,26,28,29], *Trigonella foenum-graecum *L. [7,16-18,28,29] and *Rosa canina *L. [7,16-18,28,29]. Many of these species (*Daucus carota *L., *Cynara cardunculus *L., *Allium cepa *L., *Prunus persica *(L.) Batsch., *Capsicum annuum *L. and *Lycopersicum esculentum *L.) could be considered nowadays the so -called "nutraceuticals", foods able to protect from or even to ward off several illnesses related to bad food habits.

### 12- Genital system

The 50 species involved in the reproductive apparatus act mainly in female troubles: many of them are emmenagogue or abortive (respectively 11 in Tunisia and 15 in Italy) or are used in regulating or in soothing painful menstruation. There are species that facilitate delivery, such as *Corydothymus capitatus *(L.) Reichenb. Fil. [16-18,28,29] and *Linum usitatissimum *L. [16-18,28,29], or that stop post-partum haemorrhage as *Vitis vinifera *L. leaves [16-18,28,29]. *Petroselinum crispum *L. is used to stop lactation in both countries [16-18,27-29,40,41,47], whereas *Carum carvi *L. [9-11,21,25,26] and *Pimpinella anisum *L. [7,16-18,26,29,41] are used to increase it. While *Capparis spinosa *L. is employed as galactogenous exclusively in Tunisia [16-18,28,29], *Marrubium vulgare *L. [37] is used in Italy for the same employment. Several important diseases are treated by Tunisian popular remedies: gonorrhea by *Ajuga iva *(L.) Scrheb. [16-18,28,29] (aerial parts), *Cynodon dactylon *L. (roots), *Zea mays *L. (stigmata) [16-18,28,29]. The Tunisian use of *Daucus carota *L. against orchitis is probably ascribed to a generical antiinflammatory effect [16-18,28,29] as well as the effect of *Glycirrhyza glabra *L. against prostatic adenoma in Italy [12].

### 13- CNS

Only 10 species are considered for CNS troubles. Many of them, considered sedative in Italy, are absent, not known or not used at all in Tunisia and vice-versa. Only *Papaver rhoeas *L. [7,10,14,16-18,20,21,23-25,28-30,32,33,39,45,48] and *Verbena officinalis *L. [16-18,26,28,29,40] have the same use in both countries. The use of *Peganum harmala *L. in Parkinsons' disease is noteworthy.

**Figure 2:**
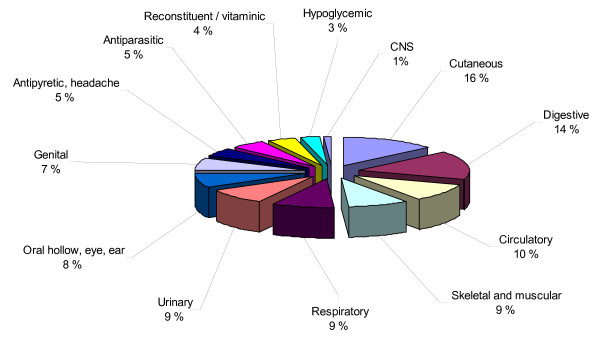
Percentage of uses in the different systems

From the recorded data it is possible to make some observations:

The more numerically present and more used families are: *Cruciferae *(16 species), *Compositae *(15), *Umbelliferae *(10), *Labiatae *(9), *Leguminosae *and *Gramineae *(5), *Rosaceae *and *Liliaceae *(4). Independently of the number of species, it is noteworthy that in the family of *Lamiaceae *every species is present at least in 5 systems (*Mentha *sp. pl., *Lavandula stoechas*) or in 9 systems (*Teucrium capitatum*) or even in 11 (*Marrubium vulgare*), while for *Compositae *family, species are utilized in 6 systems (*Artemisia absinthium *and *A. campestris*) and for *Cruciferae *no more than 5 systems (*Carum carvi*) [Figure 2]. On the contrary, the few species of *Liliaceae *play a role in several important diseases as hypotensive, hypoglycemic, cardiotonic etc. (*Allium cepa, A. sativum, Urginea maritima*).

Several species are really considered as "*panacea*" and it would be interesting to carry out further phytochemical investigations to validate these empirical uses e.g. *Ricinus communis *L. which acts (according to different used parts) as purgative, antitussive and in rheumatic pains, acne, and as corn plaster.

**Figure 3:**
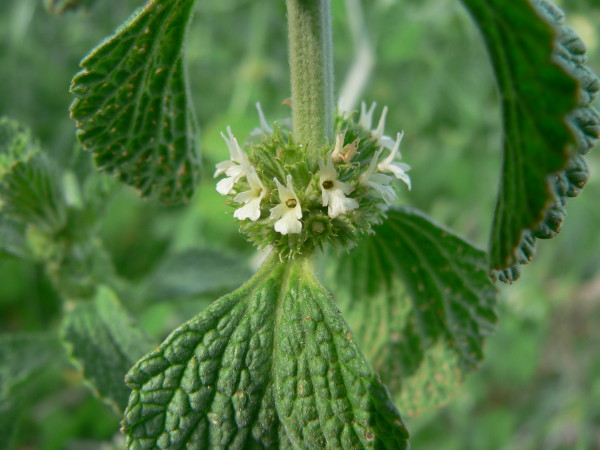
*Marrubium vulgare L*., one of the most used species in both countries

*Marrubium vulgare *L. [Figure 3] has 21 different uses in Italy and 18 in Tunisia, covering a large range of troubles and also important diseases (cardiotonic, antimalarial, hypoglycemic, hypotensive, anti decays etc...), as well as *Artemisia absinthium, Rosmarinus officinalis *(9). While *Peganum harmala *L., *Ajuga iva *Schreb., *Nigella damascena *L., *Rubus ulmifolius *Schott. and *Lavandula stoechas *L. are largely used for the treatment of a great range of diseases (8). Among the 153 species, seventy have similar uses in both countries.

Toxic species such as *Ecballim elaterium *(L.) A. Richard, *Bryonia dioica *Jacq., *Ranunculus *sp. pl., A*rum italicum *Miller, *Nerium oleander *L., *Thapsia garganica *L. etc... are also used for both internal and external uses.

It is important to underline the value of oil from *Olea europaea *L., not only as an irreplaceable and precious nutriment, but also as a good excipient which is often employed to vehicle and combine the drugs especially those used for external use. Its affinity for the skin justifies the frequent recourse to olive oil. Moreover, it is well-known since the ancient time for its important role as a protector in a large range of diseases such as liver and vessel troubles or as an anti-atherosclerosis and as hypotensive. The Tunisian use of olive oil against trachoma is noteworthy. *Olea europaea *is already mentioned in the Old and the New Testament in which it is said "*Olea prima arborum omnium est*" (that means "Olive tree, is the first among all trees") as well as in the Kuran "Olive tree is the blessing and light tree". It is noteworthy to note that even nowadays the use of olive oil in the so-called Mediterranean diet is still important.

## Conclusion

The present research shows a considerable convergence in therapeutic uses of many species belonging to Italian and Tunisian Floras, while those utilized for different purposes often bear the evidence of noteworthy and interesting properties [Table 1]. On the contrary, there are scarce different or clashing uses of medicinal plants e.g. *Globularia alypum *L. proposed for the treatment of gastric ulcer in Tunisia [16-18,29] while in Italy it is considered as purgative [12,26]; *Alnus glutinosa *(L.) Gaertn. is used as an antiulcer remedy in Tunisia [16,17] and simply as anti-inflammatory in Italy [12,14,22,26,40]. *Buxus sempervirens *L. is considered as an antineoplastic in Tunisia [11] and employed as antimalarial in Italy [7,27,37]. Sometimes different therapeutic uses of several species are related to the different considered parts (this is a consequence of different phytochemical composition) e.g. *Cuminum cyminum *L., used in Tunisia as a decoction of unripe fruits, acts as abortive, while in Italy only the decoction of ripe fruits is used as bitter tonic and carminative. *Ricinus communis *L. whose Italian use envisages only the oil from the seeds as purgative or externally as cosmetic, whilst in Tunisia the drug obtained from leaves is used in respiratory troubles, rheumatic pains and even against acne.

**Table 1:** Interesting uses

Gonorrhea	*Petroselinus crispus *L., *Cynodon dactylon *(L.) Pers., *Zea mays *L., *Ajuga iva* (L.) Schreb.
Orchitis	*Daucus carota *L.

Trachoma	*Marrubium vulgare *L., *Nigella damascena *L., *Olea europaea *L., *Capsicum annuus *L. (this last in cattle and sheeps)

Ocular leukoma	*Sonchus oleraceus *L., *Reseda alba *L., *Ficus carica *L., *Carthamus tinctorius *L.

Hemiplegia	*Vinca minor *L., *Allium cepa *L.

Hypotensive	*Pistacia lentiscus *L., *Marrubium vulgare *L., *Peganum harmala *L., *Prunus persica *L.

Measles	*Linum usitatissimum *L., *Apium graveolens *L.

Anti neoplastic	*Xanthium strumarium *L., *Buxus sempervirens *L.

Whooping cough	*Raphanus sativus *L.

Antimalarial	*Artemisia absinthium *L., *Buxus sempervirens *L., *Globularia alypum *L., *Marrubium vulgare *L.

Asthma	*Lavandula stoechas *L., *Pistacia lentiscus *L., *Rosmarinus officinalis *L., *Linum usitatissimum *L., *Polygonum aviculare *L., *Rumex tuberosus *L.

Jaundice	*Ecballium elaterium *(L.) A. Richard, *Marrubium vulgare *L., *Rumex tuberosus *L.

Anti Parkinson	*Peganum harmala *L.

Herpes zoster	*Vitis vinifera L*.

Psoriasis	*Verbascum sinuatum *L., *Ecballium elaterium *(L.) A. Richard

Erysipela	*Solanum nigrum *L., *Sambucus nigra *L.

Hydrops	*Fumaria officinalis *L.

The Resolution of the World Health Organization issued in 1990 states that " The use done for a long period of time with traditional plant base could give important information about the pharmacological effects on the Human being since the plant remedies have been used for a long time." [49]

This comparative analysis strengthens the firm belief that ethnobotanical findings represent an important shared heritage, coming over the centuries, that must not be relegated to a narrow, historical and cultural context, but gives a considerable mass of data to be still exploited in order to provide further new and useful knowledge.

## Competing interests

The authors declare that they have no competing interests.

## Authors' contributions

MLL carried out the text of the manuscript which was completed by KG. Additional file 1 was filled by both authors: MLL for the Italian ethnobotanical data and KG for the Tunisian ones. The other additional files were elaborated by both authors who read and approved the manuscript.

## Supplementary Material

Additional file 1 **Results of the comparative study.** Click here for file
