# Supplementary material for: Comparative analysis of medicinal plants used in traditional medicine in Italy and Tunisia
Source: J Ethnobiol Ethnomed. 2009 Oct 26;5:31. doi: 10.1186/1746-4269-5-31 (PMC2773757; doi:10.1186/1746-4269-5-31)
Supplement: Additional file 1 — Results of the comparative study. [file 1746-4269-5-31-S1.PDF]

Additional file 1. Results of the comparative study

Botanical name and Family	Tunisian name	Italian name	Tunisian use	Italian use	Part used in Tunisia	Part used in Italy	Tunisian manipulation	Italian manipulation	Notes	Bibliographic References
**1 Adiantaceae**										
Adiantum	Chojret	Capelvenere	**Cough,**	**Antitussive**	Whole plant	Aerial part	Infusion	Not specified	abandoned In Italy	7,9,13,14,18,25,26,27 28,31,39,41,43
capillus - veneris L	el bir,		gastralgia	Lenitive,						
.	Maadnous			expectorant						
	el bir			Emmenagogue						
				Antalgic in		Aerial part		Cataplasm	Only in	
				delivery pains				Decoction	Sardinia	
				Diuretic						
				Tooth ache		Leaves		Powdered leaves		
								locally applied		
**2 Anacardiaceae**										
Pistacia lentiscus L.	Dharw	Lentisco	Gastralgia	Astringent	Leaves	Resin	Decoction	In pills		7,12,13,16,17,18,26, 27,28,29,31,39,41, 43,45,47,
			Hypotensive	Haemostatic	leaves	exudating	To chew			
			Pyrosis		leaves	from	To be eaten			
					and fruits	the bark				
			**Antitussive**	**Expectorant**			Decoction			
			Asthma							
			**Rheumatism**	**Rheumatism**	Oil from the fruit	Leaves	Oil locally	Cataplasm locally applied (in		
				Arthritis		Stem	applied	mixture		
								with other plants)		
			**Dental pain**	**Antiodontalgic**		Stem	Decoction	To chew		
			**gingivitis**				as mouth wash			
				Antidiarrhoeic		Resin		Infusion		
				Cicatrizant		Bark				
				Burns						
				Swollen feet		Leaves		Into the shoes		
				Antipyretic		Leaves		Decoction		
				Fight against scabies		Oil from		Locally applied		
						Fruits				
Pistacia	Battoum	Terebinto	see *P. lentiscus*	Expectorant	Leaves	Resin from	Decoction	As ointment		13,14,16,17,26,28,29, 39
terebinthus L.				Vesicatory		the bark				
Pistacia vera L.	Fostok ,	Pistacchio	Gastralgia	Alimentary	Fruits	Seeds	to eaten	To be eaten as snacks		7,9,26,29
	fosdok			Lumbago		Resin locally				
						Rubbed				
**3 Apocynaceae**										
Nerium oleander L.	Defla	Oleandro	Antigangrenous	Cardiotonic, Fight against itching	Leaves	Dried leaves	Powdered	Powdered	toxic plant	7,12,16,17,18,24,25, 26,28,,29,36,,43
							and locally	leaves in		
				Smokede as		Dried	applied	infusion		
				antiasthmatic		flower		for itching		
				Against scabia		Flowers		Poultice of		
								flowers		
								mixed with		
								honey		
			Dermatosis		Stems		Rubbing	(only in e.u.)		
			eczema							
			sores,		Leaves		Poultice			
			sunburns							
			Scalp stimulating							
				Antiasthmatic		Dried		To smoke		
						flowers			Only in the	
									Sorrentina	
**4 Araceae**										
Arum italicum	Sabbat el	Aro, Gigaro	Antiseptic	Antirheumatic	Leaves	Tuber	External use	Tincture	Toxic plant	7,16,17,18,26,27,28, 29,40,43,47,48
Miller	ghoula		Antibacterial	Revulsive	.		locally			
				Anti-inflammatory		Fresh plant	applied	Squashed and		
				Haemorrhoids		Leaves		locally applied		

				Antirheumatic		Rhizome		Ground plant part (e.u.)		
				Chilblains						
**5 Araliaceae**										
Hedera helix L.	Chaabata, Mahboula	Edera	**Emmenagogue**	**Emmenagogue**	Leaves	Leaves	Decoction	abandoned		7,9,10,12,14,18,20,22 23,24,25,26,27,28,29, 31,35,40,41,45,48
			Anti spasmodic	Gall stones		Young leaves		Infusion		
				Toothache				decoction		
				Expectorant		Leaves		abandoned		
				Antineuralgic				for its toxicity	Fruits can cause severe allergies	
				anesthetic in rheumatic and arthritic pains (only e.u.)						
				Cicatrizant of sores and Varicose ulcers and haemorrhoids				Infusion as bath foot		
				Antiflogistic		Fresh plant		Squashed		
				For corns and warts		or leaves		and locally		
				Lenitive for the skin		Leaves		applied		
				To darken hair		Leaves		Decoction as shampoo		
**6 Aspidiaceae**										
Dryopteris filix-mas (L.) Schott	Not reported	Felce maschio	**Antihelmintic**	**Antihelmintic** (mainly against *Taenia solium)*	Aerial parts	Rhizome	Decoction	Decoction		7,10,18,24,26,27,28, 29
				Antalgic for swollen feet and ankles		Rhizome		Decoction in vinegar as foot bath		
**7 Betulaceae**										
Alnus glutinosa (L.) Gaertn.	Oud el ahmer	Ontano	Antipyretic	Mouth wash, anti- inflammatory	Bark	Bark	Decoction locally applied	Decoction		12,14,16,17,26,40
			Headache							
			Gastralgia	Vulnerary		Bark				
			Antiulcer	Astringent		Sap from the stems	Decoction	Locally applied		
				Anti parasitic (mainly fleas)		Leaves		Leaves scattered on the floor		
**8 Boraginaceae**										
Alkanna tinctoria (L.) Tausch	Hennet el ghoula, Chenjar	Alcanna	**in dyeing clothes**	**in dyeing clothes**	Roots	Roots	Decoction	Decoction		7,12,18,28,29 ,
Borago officinalis L.	Boukhrich Boukrich,	Borraggine	**Emollient**	**Emollient** lenitive for the skin,	Leaves	Leaves flowering tops	Decoction	Infusion, Decoction		9,10,11,13,14,16,17, 18,20,22,24,26,27,28, 29,30,31,31,32,34,35, 40,41,42, 43,47,45,48
			**Antitussive**	**Antitussive**						
				Expectorant Asthma		Leaves		To eaten boiled		
			**Diuretic**	**Diuretic**	Leaves	Fresh plant	Decoction	Decoction or Infusion		
				Mild laxative		Juice from the leaves and stems		to swallow		
				Depurative		Leaves		Decoction mixed with *Urtica* sp.pl.leaves		
			Diaphoretic		Leaves		Decoction			
Heliotropium europaeum L.	Dhaharet el chems	Erba porraia	Toxic	Astringent vulnerary	Generally not used for its toxicity	Aerial parts, roots, seeds	Not used since its toxicity	Infusion, decoction		7,13,26,27,28,29
**9 Buxaceae**										

Buxus sempervirens L.	Beuqs	Bosso,<br>Bòssolo	Antineoplastic	Laxative, cholagogue<br>Antipyretic<br>Antimalarial	Not specified	Leaves,<br>bark	Not<br>specified	Infusion,<br>decoction	The plant is toxic enough and must be used in low dose and very cautiously - Fresh leaves are used in adulterating of the leaves of *Arctosta-phylos uva-ursi* and also in aromatizing beer	7,18,20,27,29,37, 45,48
**10 Capparaceae**										
Capparis spinosa L.	Kabbar	Cappero	Headache	Diuretic	Leaves	Bark	Cataplasm	Decoction	Buds are eaten for their hot taste in aromatizing foods	9,16,17,18,23,25,26, 27,28,29,30,34,48
			**Antirheumatic**	**In artrithic pains**						
				Digestive		Flower buds		To be eaten		
			Cold, sinusitis	Liver diseases			Decoction			
			Hypotensive							
			Galactogenous				Cataplasm			
				Antiscorbutic		Flowers buds		To eaten mixed with vinegar		
				Varicose ulcers				squashed and locally applied		
				Cicatrizant		Flower buds		Squashed (e.u.)		
**11 Caprifoliaceae**										
Sambucus ebulus L	Khelouan sghir	Ebbio	**Diuretic**<br>Laxative	**Diuretic,**<br>Laxative	Leaves	Roots, fruits, flowers	Decoction	Decoction, infusion		10,18,24,26,28,29, 45
			**Antirheumatic**	**Antirheumatic**<br>antineuralgic						
Sambucus nigra L.	Okkez sidi moussa,	Sambuco	**Diuretic**	**Diuretic**,	Leaves	Flowers,	Infusion	Infusion,		7,10,12,14,16,17,18, 21,22,23,25,26,27,28, 29,30,35,40,42, 43,47, 45,48
			Antiseptic	laxative		fruits	Decoction	Decoction		
			**Emollient**	**Emollient**		Leaves	Decoction	Squashed leaves		
				Antipyretic			Infusion	locally applied		
			**Antirheumatic**	**Antirheumatic**		Bark,	Pounded with fat	Decoction		
			Diaphoretic	antiarthritic		fruits or	Infusion	Juice from		
				antineuralgic		leaves				
				(sciatic and			Locally applied	squashed fruits		
				trigeminal nerve)						
				Analgesic			Warmed leaves			
				Toothache		Leaves		Decoction		
				Eye inflammation		Flowers				
			**Emollient**	**Emollient,**	Leaves	Leaves	Decoction	Decoction		
				antitussive,		Inflorescence		Decoction		
				expectorant						
				Antiasthmatic		Leaves		Infusion		
				Anticholesterol						
				Distorsions (sprains)		Leaves		Squashed and boiled then locally applied		
				Cystitis		Dried bark		Decoction		
				Eye wash		Flowers,		Decoction		
				Antipyretic		Fruits				
						Bark		Decoction		

				Varicose veins		Leaves		Cataplasm of fresh ground leaves		
				Cicatrizant						
**12 Cesalpinaceae**										
Ceratonia siliqua L.	Kharroub	Carrubo	**Antidiarrheal for children**	**Antidiarrheal for children**	Fruit	Pulp, Seeds	Flour from dried and crushed pulp	Decoction		7,9,10,13,14,16,17,18 26,28,29,31,32
			Hypoglycemic	Emollient for the skin, cosmetic	Fruit		Cataplasm Pulp from dried and			
			Heart diseases	Cough, catarrh	Fruit	Seeds	crushed seeds	Decoction		
			Inflammation of oral cavity		Fruit					
**13 Chenopodiaceae**										
Chenopodium ambrosioides L .	Lajouma	Tè di Germania	Gastralgia and aerophagia of infants	Antihelmintic		Flowering tops		Infusion	Abandoned	7,17,18,25,26,27
			**Carminative**	**Carminative** Diuretic						
Salsola kali L.	Kali	Erba soda, Riscolo	Hypotensive	Source of mineral Elements : Na, Oxalate, etc. Diuretic in kidney stones	Leaves	Leaves	Not specified	Infusion		12,26,29
Spinacia oleracea L .	Sebnakh	Spinacio	**Alimentary**	**Alimentary** Laxative	Leaves	Leaves	Not specified	Fresh or boiled the leaves are eaten as vegetable	The plant is rich in Fe	7,12,26,27,29
				Cosmetic as face mask				Juice from fresh leaves		
**14 Compositae**										
Artemisia absinthium L. .	Chojret mariem	Assenzio	**Antihelmintic**	**Antihelmintic** Diuretic Kidney stone	Leaves	Aerial parts	Decoction	Decoction	Leaves and flowers are used to Aromatizer for liqueurs	7,16,17,26,27,28,29, 48
			**Abortive** **Contraceptive**	**Emmenagogue**	Leaves	Leaves	Decoction	Decoction		
				Antipyretic Antimalarial		Aerial parts		warmed in wine		
			Anti-ulcerous and gastralgia	Antiseptic Mouth-wash	Leaves		Decoction	then filtered		
			Antitussive	Toothache		Aerial parts		Tincture (as imbued cotton flock) (only in e.u.)		
			Cardiac analeptic							
Artemisia campestris L. .	Tgouft, Chaal	Not specified	Antivenomous in insect bites	Antipyretic	Leaves	Aerial parts	Powder	Decoction		16,17,18,26,28,29
			Cicatrizant Anti-ulcerous Antitussive				Decoction			
			**Hypoglycemic**	**Hypoglycemic** Anticholesterol		Dried flowering tops		Infusion		
			**Cicatrizant**	**Cicatrizant**	Leaves	Aerial parts	Powder	Decoction (imbued compresses)		
Calendula arvensis L.	Karchoun, Llouach	Fiorrancio	Antirheumatic Antipyretic Headache Hypotensive	Lenitive for the skin, Detergent Cicatrizant	Leaves, flowers fruits	Capitula (heads), Leaves	Cataplasm Cataplasm Cataplasm Decoction	Infusion		7,11,12,13,16,17,18, 20,24,25,26,27, 28,29,31,41,48
			**Emmenagogue**	**Emmenagogue**	Aerial parts		Decoction			
				Warts and corns		Leaves		Juice from the leaves locally applied		

				Gallstones		Aerial parts		Decoction		
Carthamus tinctorius L.	Zaafrane	Zafferano	Leukoma	Revulsive in	Leaves	Oil from	Not specified	external use		17,28,26
		bastardo	dysmenorrhea	painful joints	Flowers	seeds	Maceration			
			in menstrual pain				in olive oil			
			Eye wash				Decoction			
Centaurea calcitrapa L.	Bou naggar,	Calcatrppola	**Antipyretic**	**Antipyretic**	Aerial parts	Flowering	Decoction	Infusion		7,16,17,18,28,29,45
	Bou chouika		In cattle jaundice	Antimalarial	Roots	tops	Maceration			
				Tonic, digestive			in water			
				Antiodontalgic		Whole plant		As gargling		
Cichorium intybus L.	Chikouria,	Cicoria	**Hypoglycemic**	**Hypoglycemic**	Leaves	Roots,	Fresh leaves	Decoction,		7,9,14,16,17,18,20,22, 23,24,25,26,27,28,29, 30,35,40,41,42, 43,47 48
	Skouria					Aerial parts	to eaten			
			**Diuretic**	**Diuretic**		Leaves		Fresh leaves		
								boiled in milk		
				Cholagogue				squashed		
				stimulant of gastric secretion				fresh leaves		
				Kidney trouble		Root		Decoction		
				Antianemic		Leaves		Decoction		
				Jaundice						
				Liver protecting						
			**Laxative**	**Laxative**						
				Cicatrizant for		Leaves		Infusion (e.u.)		
				sores and burns						
Conyza	Jaada	Saeppola	**For rheumatic**	**For rheumatic**	Flowering	Fresh juice	Decoction	Infusion		17,26
canadensis (L.) Cronq			**pains**	**pains and gout**	tops					
(= *Erigeron conyza.* L.)			Gastric ulcer	Astringent		Aerial part	Decoction	Infusion		
			gastralgia	intestinal						
				Diuretic						
				anticholesterol		leaves		Decoction		
			Cardiac analeptic	In bronchial		Fresh juice				
				catarrh						
			**Tonic**	**Tonic**	Flowers	leaves	Powder	Decoction		
							mixed			
							with honey			
Cynara	Khorchef,	Carduccio	Kidney stones	Energetic	Capitula	Capitula	Decoction	To eaten raw		7, 9,15,16,17,18,20,21 25,26,27,28,29,30,39, 41,42,43
cardunculus L.	Kardhoun			Liver protecting	(heads)	(heads)		or boiled		
				Depurative of blood		Leaves				
.			Haemorrhoids	Source of Fe	Flower, roots	Leaves	Decoction			
				Stomach ache		before		Infusion		
			Choleretic		Flower, roots	flowering	Decoction			
			Cardiac analeptic		Flower		Decoction			
				Hypogicemic		Roots	Decoction			
Cynara cardunculus L. subsp.*scolymus* (L.) Hayek	Guennaria	Carciofo	**Choleretic,**	**Choleretic,**	Leaves	Roots,	Decoction	Decoction	The unripe heads are eaten	7,9,10,16,17,21,23,24 26,27,29,40,43,48
			gastralgia	cholagogue		leaves				
			Kidney stones	Cosmetic as	Capitula		Fresh bud			
				face mask	(heads)	leaves	to eaten	Infusion		
			**Liver protecting**	**Liver protecting**		Leaves				
				Gallstones		Leaves		Infusion		
Helianthus annuus L.	Ain el	Girasole	Sunstroke	Dietetic,	Capitula	Seeds	Maceration	Oil	raosted seeds	7,17,18,27,29
	chems			promoting		Seeds	in warm oil		are enjoyed	
				digestion			locally		as snacks.	
				anticholesterol			applied			
				Diuretic			on the head			
				Mild sedative		Seeds			Oil is used	
				Hypotensive					in cooking	
				Antipyretic		Leaves		Alcoholic		
				Hypoglycemic				maceration		
				Lenitive for		Flowers		Infusion		

				the skin						
Matricaria chamomilla L.	Babounej, Bibounej	Camomilla	**Gastralgia**<br>Aerophagia<br>nephritic colic<br>Depurative<br>Fight against post- partum pains	**Gastralgia**<br>Emollient<br>soothing and cicatrizant<br>For the skin<br>Mild sedative<br>Eye wash<br>Oral hollow inflammation	Whole plant<br>Capitula (heads)	Capitula (heads)	Decoction<br>Decoction	Infusion		7,10,15,16,17,18,23, 24,25,26,27,39,40,41, 42, 43,47,45,48
Senecio vulgaris L.	Jedla, Jedia	Calderugia, Verzellina	**Emmenagogue**<br>Uterotonic	**Emmenagogue**<br>Astringent<br>Diuretic<br>Antihelmintic mainly for intestinal worms<br>Varicose veins	Not specified	The whole plant	Not specified	Infusion	Toxic plant due to the presence of senecionine	7,11,18,23,24,26,28, 29,31
Silybum marianum (L.) Gaertn.	Chouk el jmel	Cardo mariano	Antipyretic<br>Anti-haemorrhagic	Choleretic, cholagogue., liver protecting<br>Diuretic	Capitula	Capitula (heads)<br>Roots and fruits<br>Leaves	Decoction	Decoction<br>Decoction<br>Infusion		7,12,18,26,27,28,29
Sonchus oleraceus L.	Difef, Tifef	Cicerbita	Treatment of verrucae (warts)<br>Ocular leukoma	Cholagogue cathartic<br>Gastralgia<br>Cicatrizant	Flowering tops<br>Leaves	Leaves<br>Leaves	Juice locally applied<br>Juice locally applied	eaten as vegetable<br>Juice from the chewed leaves or infusion<br>Cataplasm from fresh squashed leaves		7,12,15,17,27,28,43, 47,45,48
Xanthium strumarium L. (= *X .s.* var. *brasilicum* (Vellozo) Fiori	Akkar	Lappola	Anti - neoplastic<br>Depurative, **diaphoretic**<br>Stomachic, emmenagogue	Diuretic,<br>**diaphoretic**<br>breathing ailments	Not specified<br>plant	The whole	Not specified	Decoction		26,28,29
**15 Convolvulaceae**										
Convolvulus arvensis L.	Alleg, Lawaya	Vilucchio	Hair stimulant	Laxative<br>Diuretic<br>Furuncolosis<br>Antihelmintic<br>Liver protecting	Bark, stems	Aerial part, root<br>Leaves<br>Leaves, root	Locally applied	Tincture,<br>Infusion,<br>Cataplasm<br>Infusion		7,10,17,26,28,29,31, 43,45,48
**16 Cruciferae**										
Brassica napus L.	Left	Ravizzone, Navone	Antipyretic<br>Improve visual acuteness	Revulsive	Roots	Seeds	To eaten fresh	Decoction		15,16,17,26,28,29
Camelina sativa (L.) Crantz	Hanjaret el koubaa	Camarin, Camarina	Alimentary	Emollient, lenitive, astringent<br>Cicatrizant	Not specified	Seeds<br>Leaves	Not specified	Decoction<br>Locally applied		26,29
Capsella bursa-pastoris (L.) Medicus	Kiss er raai	Borsa del pastore	**Haemostatic**	**Haemostatic,** Diuretic, Emmenagogue<br>Hypotensive	Not specified	Whole plant	Not specified	Decoction, Juice of fresh plant, or dried powdered plant<br>To be eaten fresh		7,10,13,14,18,20,25, 26,27,29,45,48

				Impetigo,<br>furunculosis		Fresh plant		Ointment<br>from fresh plant<br>mixed with<br>oil or fat		
Nasturtium officinale R.Br.	Hab erched	Crescione d'acqua	**Antiscorbutic**<br>**Diuretic**	**Antiscorbutic**<br>Digestive<br>Depurative<br>Eupeptic<br>**Diuretic**	Seeds	Aerial part	To eaten as food	Fresh plant is eaten as salad<br>Infusion		7,18,26,27,28,29,45
Raphanus raphanistrum L	Fjel el jabi	Rapastrello, Ramolaccio	Alimentary	Fight against gall-stone, liver protecting<br>Diuretic<br>Antitussive<br>Gingivitis	Root,	Root, juice of fresh plant	To eaten as food	Decoction<br>As mouth-wash		7,11,27,29
Raphanus sativus L.	Fjel	Ravanello	Antitussive mainly<br>**Whooping cough**	Stimulating cutaneous blood-flow<br>**Whooping cough**	Root	Root	hollowed out heart Radish imbued with Sugar; after 1 day, sugar is eaten fresh	Raw roots are eaten mixed With salad<br>Chopped and flavoured with honey		7,12,17,18,28,29
Sinapis alba L.	Khardhal abiadh	Senape bianca	**Rheumatic pains**<br>**Flavouring for Foods**	**Rheumatic pains** for catarrh revulsive (only e.u.)<br>**Flavouring for foods**	Seeds	Seeds	Not specified	Cataplasm with flour from ground and warmed seed		7,18,26,27,28,29
Sinapis arvensis L	Khardhal	Senape selvatica	Rheumatic pains<br>Antitussive	See *S. alba*	Leaves<br>Leaves added with Lin *(Linum usitatissimum)* seeds	See *S. alba*	Boiled leaves, reduced as paste mixed with vinegar<br>Cataplasm on chest	See *S. alba*		7,16,17,26,
**17 Cucurbitaceae**										
Bryonia dioica Jacq.	Qria	Vite bianca	Not used	Purgative<br>Diuretic<br>Antitussive<br>Painful articulations<br>Furunculosis	Not specified	Leaves<br>Young stem leaves and berries<br>Leaves (e.u.)	Not specified	Infusion<br>Infusion<br>Cataplasm of fresh leaves	Toxic plant	7,10,17,18,26,28,29, 42,45,48
Citrullus colocynthis (L.) Schrader	Handhal, Dellaa el oued	Coloquin-tide	Impetigo, cicatrizant for injuries and wounds<br>Rheumathic pains<br>Hypotensive<br>Gynecological diseases<br>Haemorroids	Strong purgative<br>Emmenagogue<br>Abortive	Pulp of the fruits<br>Fruits<br>Seeds<br>Dried fruits	Pulp of the fruits	Warmed pulp locally applied<br>Crushed and warmed fruit locally applied<br>Decoction in water and oil locally applied<br>Decoction<br>Mixed with honey	Extract	Abandoned since its toxicity	16,17,18,26,28,29
Cucurbita pepo L.	Kraa	Zucca	Tinea<br>(sunstroke ,	Burns<br>Laxative<br>Mastitis	Leaves	Fruits	Applied on shaved head<br>fruits applied	Squashed pulp of fruits locally applied	More uses as food	7,11,17,18,21,29,48

			typhoid)				on head			
				Antihelmintic		Peeled seed macerated in water		The paste mixed with sugar and honey is eaten , after It is necessary to take a purge		
				Prostatic trouble		Dried or peeled seeds		As preventive		
Ecballium elaterium (L.) A. Richard	Faquous h'mir Faquous el bhaiem	Cocomero asinino	**Drastic purgative**	**Strong purgative**	Fruits	Fruits,	Juice mixed with tobacco	Infusion		7,8,12,13,16,17,18,26, 28,29,31,48
			Sinusitis migraine	Psoriasis	Fruits	aerial parts Roots		Decoction evaporated then filtered in mixture with *Verbascum sinuatum*, and *Verbena officinalis*		
			Spot pimple		Fruits		Crushed fresh fruit Locally applied			
			**Jaundice**	**Jaundice**	Fruits			Juice of unripe fruit instilled into eyes		
**18 Cupressaceae**										
Cupressus sempervirens L.	Sarwel	Cipresso	**Haemorrhoids**	**Haemorrhoids**	Leaves	Cones	Decoction	Extract	Largely used as ornamental	7,12,16,17,18,20,26, 27,28,29
				Astringent for blood vessel						
			Gastralgia, stomach pains	The essence is antitussive breathing ailments		Young stems		Infusion		
Juniperus oxycedrus L.	Taga, Araar	Ginepro rosso	Antidiarrhoeic	Against skin parasites	Leaves	Oil of "Cade" fruit and wood	Decoction	External use		7,12,13,14,16,17,18, 26,28,29,48
			Hypoglycemic Cicatrizant vulnerary		oil of "Cade"		Locally applied			
				Lumbago and sciatic nerve pains		Fresh stems		Decoction as bath		
				Diuretic Digestive Expectorant		Pseudofruits		Infusion or syrup		
				Stomach pains		Pseudofruits		Decoction		
Juniperus phoenicea L.	Araar	Ginepro fenicio	**Abortive** Emmenagogue oxytocic Antirheumatic Hypoglycemic	**Abortive**	Leaves	Young stems	Decoction	abandoned illegal use in Italy		12,16,17,18,26,28,29
**19 Cyperaceae**										
Cyperus longus L.	Saada	Zigolo	**Cardiac diseases**	**Cardiac troubles**	Roots	Rhizome	Ground roots to eaten in small amount		*C.esculentus L.* more used in Italy	16,17,26
				Refreshing		Oil from rhizome				
**20 Dioscoreaceae**										

Tamus communis L.	Karma souda	Tamaro,<br>Vite nera	Antirheumatic	Diuretic,<br>Anti-inflammatory in urinary diseases	Roots	Roots	External use	Decoction,		7,13,18,26,28,29
				purgative/laxative				Tincture		
				Emetic				Extract		
			**Ecchymosis**	**Ecchymosis**<br>Bruises, Rheumatic, arthritic and lumbago pains		Pseudofruits		Squashed and locally rubbed		
**21 Equisetaceae**										
Equisetum telmateja Ehrh<br>(=*E. maximum* Lam.)	Dhenb el Khil,<br>Dhenb el Fars	Coda di cavallo	**Diuretic**<br>**Hemostatic**<br>mineralizing	**Diuretic,**<br>**Hemostatic**<br>Restructurent of lung cartilage	Not specified	Young stems	Not specified	Infusion	Unadvisable for people with renal diseases	7,9,11,22,26,27,28,30 35,37,47,48
				To strengthen nails and hair<br>cicatrizant of sores<br>Source of microelements					For its content in Si	
**22 Ericaceae**										
Arbutus unedo L.	Lenj,<br>boujbiba	Corbezzolo	Hypoglycemic	Astringent<br>Antirheumatic	Leaves	Leaves,	Decoction	Infusion	Fruits are employed in	7,11,16,17,26,27,28, 29,45,48
				Diuretic,		Fruits		Infusion	preparing	
			Hypotensive	renal trouble	Roots	Root	Decoction		homemade	
			**Antidiarrheal**	Liver protecting	Seeds	Bark	Decoction	Decoction	jams and	
				Anti-atherosclerosis		Fruit	Decoction	Decoction	soft	
				Cicatrizant		Bark	Decoction	Locally applied	liqueurs	
				**Antdiarrheal**		Leaves	Decoction			
Erica arborea L.	Bouhaddad	Erica	**Urinary antiseptic**<br>Astringent	**Diuretic**,<br>Antirheumatic	Flowering tops	Flowering tops	Not specified	Infusion,<br>decoction		7,12,16,26,28,29,45, 48
**23 Euphorbiaceae**										
Mercurialis annua L.	Hbaq eddhol,<br>H'biquet eddhol	Mercorella	**Diuretic**	**Diuretic**	Leaves	Leaves	Decoction	Infusion	Toxic plant	16,17,18,26,28,29,37, 43,45
			Leukorrea	Renal stones						
			Frigidity	Nephritic colic						
			**Purgative**	**Laxative**	Leaves	Leaves	Decoction	Infusion (as enema)		
				Cholagogue, choleretic		Leaves		Decoction		
				Stops milk secretion		Whole plant		Decoction		
Ricinus communis L.	Kharwaa	Ricino	Bechic<br>Bronchitis<br>Headache	Strong purgative	Leaves	Oil from peeled seeds	Leaves imbued in olive oil applied on thorax and back	one or two spoon with oil to swallow		7,12,16,17,18,23,28, 29,48
				To strengthen hair (e.u.)		Oil from peeled seeds		Oil locally applied		
			Fever<br>Rheumatic pain	Corn plaster<br>Emollient for the skin	Leaves		Fresh crushed leaves in cataplasm			
			Spot, acne	Fester	Leaves	Oil from seeds	Fresh crushed leaves mixed with vinegar in cataplasm	Cataplasm with leaves of *Verbascum sinuatum* L. mixed with castor oil		
**24 Fagaceae**										
Quercus sp. pl.	Ballout<br>(in Tunisia : *Q.ilex* L.)	Quercia<br>In Italy several	**Hemorrhoids**	**Hemorrhoids**<br>Hemostatic	Stem bark	Bark, galls	Dried and ground	Decoction		7,16,17,18,22,24,26, 28,29,37,40,41,42, 43, 45
				Antiacne		acorn	mixed with honey	of acorns flour		
				Astringent		Bark				

		different species are similarly used		Mouthwash (as antiseptic)		Leaves and stem sap		Sliced young Stem, dropping sap, locally applied	
				Burns		Bark		Decoction	
			Tonic	Antirheumatic	Stem bark		Dried and		
				Children catarrh and cough		Acorns or leaves	ground mixed with honey	Decoction	
			**Antidiarrheal**	**Antidiarrheal**		Acorns		Powder from roasted acorns	
				Gingivitis and decay		Bark		Decoction as mouthwash	
				Vaginal discharge				Decoction as washing	
**25 Fumariaceae**									
Fumaria Capreolata L.	Sibana, Hichichet essiben, Baouala	Fumosterno bianco	Spot and crusts of children scalp	Laxative, Diuretic bitter tonic	Flowering tops	Flowering plant	Crushed flowering tops locally applied	Infusion	7,12,17,22,28,48
			**Depurative**	**Depurative**	Whole plant	Flowering plant	Decoction	Infusion	
			Treatment of spots	Against alopecia				Infusion for rubbing	
			Infected injuries of ear lobe fold	To improve capillary vessel circulation	Leaves	Flowering plant	Dried powdered leaves locally applied	Infusion	
				Rheumatism and arthritis		Flowering plant		Infusion	
				Skin sores		Root		Powdered	
Fumaria officinalis L.	Sibana	Fumosterno	Dermatitis dermatosis	Laxative, Diuretic bitter tonic	Whole plant	Flowering plant		Infusion	7,12,18,23,26,28,37, 45,48
				Against alopecia To improve capillary vessel circulation Antiecchymotic		Aerial part		Infusion as washing	
				Rheumatism and arthritis		Flowering plant		Infusion	
				Hydropsy		Juice		To assume orally	
**26 Gentianaceae**									
Erythraea pulchella (Swartz) Fries (= *Centaurium pulchellum* (Swartz) Druce	Qosset sbiya, Qosset el haya	Cacciafebbre	Depurative	Digestive, Cicatrizant, Antipyretic To dye hair	Whole plant	Flowering plant	Infusion	Infusion, tincture	17,26
Centaurium erythraea Rafn. (=*C. umbellatum* (Gilib) Beck) subsp *. Centaurium erythraea subsp. suffruticosum (Griseb.) Greuter* (Salzem)	Mararet el hanech, Kosset el haya	Caccia-febbre	Toxic Bitter depurative	Digestive, tonic Gastralgia Cough, flu, antipyretic	Whole plant	Flowering plant	Infusion	Infusion, Decoction	7,16,17,18,22,25,26, 28,29,45
		Cicatrizant				Tincture			
				hair dyeing		Flowering plant		Infusion,	
**27 Geraniaceae**									
Erodium cicutarium (L.) L'Her.	Mochita, Mouchita	Cicutaria	**Astringent** Gastralgia Antidiarrheal	**Astringent** Haemostatic Diuretic	Aerial part	Aerial part	Decoction	Infusion	12,17,18,26,28,29
				For insects bites				To rub locally	
			Oxytocic				Decoction		
Geranium robertianum L.	Aterchia	Erba Roberta	Astringent Antidiarrheal	Vulnerary cicatrizant, Hemostatic	Aerial part	Aerial part	Decoction	Fresh plant locally applied	7,16,18,26,27,28,29
			Antispasmodic	throat and mouth anti-inflammatory	Leaves flowers		Internal use	Infusion as mouthwash or gargling	

**28 Globulariaceae**										
Globularia alypum L.	Zriga, selgha, taselgha	Sena di Provenza	Cicatrizant (acne, eczema, spots, abscesses) Tinea	Purgative Diuretic	Leaves	Leaves, flowering tops Resin	Powder or decoction locally applied	Infusion		1,12,16,17,18,26,29
			Gastric ulcer		Leaves		Maceration in oil			
			Antimalarial		Leaves		Concentrate decoction			
			Antirheumatic		Leaves		Concentrate decoction			
			Sore throat				Decoction as gargling			
**29 Gramineae**				.						
Arundo donax L..	Ksab	Canna	**Diuretic**	**Diuretic**<br>Diaphoretic	Aerial part	Rhizome	Decoction	Decoction		7,16,17,25,26,28,29, 32,39,41,45, 48
				Hemostatic<br>cicatrizant		Stem		Locally applied		
				Otitis		Leaves		Fumigations		
				Gastralgia		Rhizome		To eat		
Avena sativa L.<br>In Tunisia :<br>*A. flavescens* (L.) Beauv.	Not specified<br><br>Hchichet errih	Avena	Only as alimentary	Emollient for the skin<br>.		Seeds		Decoction		7,17,18,26,29,45
Cynodon dactylon L.	Njem, nejm	Gramigna, Zizzania	**Diuretic**<br>Gonorrhea<br>Antidiabetic	**Diuretic**<br>Kidney stones<br>Renal trouble	Roots	Rhizome	Decoction	Decoction from Rhizome previously macerated in Water to lose Bitter taste		7,9,11,16,17,18,20,21 22,23,25,27,28, 29,32,34,35,37,40,41 43,44, 45,47,48
				Hypotensive		Stolons		Infusion		
			**Antirheumatic**	**Antirheumatic**		Rhizome		Decoction		
Hordeum vulgare L.	Chaïr	Orzo	Urinary lithiasis	anti diarrheal<br>Anti-inflammatory for intestinal tract	Seeds	Seeds	Decoction	The water of the boiled seeds to drink		7,9,11,15,17,23,25,27 28,29,33,39,41,42,44, 45
			Galactogenous	Emollient for the skin	Seeds		Decoction	The squashed seeds previously boiled are locally applied		
			Slimming	Throat inflammations	Stems		Decoction			
				Mucolytic, cold		Aerial parts		Fumigations with Dried aerial parts		
				Cough				Decoction mixed with *Malva sylvestris* L.		
Zea mays L.	Ktania	Mais, Granturco	Laxative<br>Gonorrhoea	Diuretic<br>kidney stones	Stigmata	Beards or stigmata	Decoction	Decoction	Only in the Sardinian region	7,16,17,18,20,22,24 25,27,29,30,37,39,40 42, 44, 48
				Emollient for the skin<br>sweating promoter		Seeds		Decoction		
				Hypotensive		Stigmata (beard)		Decoction		
				Children bronchitis				Crushed seeds mixed with water such as "polenta" (pudding of maize) added with bran as cataplasm on the breast		
**30 Juglandaceae**										
Juglans regia L.	Jouza, Jouzet Essouak, Souak (root, Bark)	Noce	Toothpick	Antiseptic	Root bark	Leaves	External use	Infusion	Fruits are Employed in preparing a typical	7,16,26,27,28,39,40, 41, 45,48
				Hemorrhoids		Bark		Decoction as washing		
			Mouthwash	Mouthwash<br>Antihelmintic		Husk		Decoction		

			Hypotensive	Mouth wash	Husk		Decoction		italian<br>homemade<br>liqueur<br>"Nocino"	
			**Astringent**<br>in dermatosis	**Astringent**<br>Cicatrizant<br>for ulcers and<br>wounds infected<br>by flies	Leaves,<br>flowers	Leaves<br><br><br>Pulp of the<br>fruit "mallo"	Ointment<br>mixed with<br>grease	Juice of<br>fresh leaves<br>locally applied<br>The squashed<br>"mallo"<br>rubbed on the<br>injuried skin		
			Tonic<br>Haemostatic	For strenghtening the<br>skin of feet and<br>hands	Leaves		Decoction			
			Aphrodisiac	Fight against chilblains<br>and warts				Decoction		
				Diuretic		Leaves		Infusion		
				Antiasthmatic		Leaves		to be smoked		
				Frostbitten hands,<br>feet and ears		Leaves		decoction mixed<br>with oak bark<br>as washing		
				Acne		Leaves		Decoction for<br>fumigations		
**31 Labiatae**										
Ajuga iva (L.)<br>Schreb.	Chandgoura	Iva<br>moscata	Hypotensive<br>Respiratory<br>tract diseases<br>asthma, cough<br>Gonorrhea	Diuretic<br>Antispasmodic<br>Cicatrizant<br>Antipyretic	Aerial part	Leaves	Infusion,<br>Decoction	Infusion		16,17,18,26,28,29
			Rheumatism		Aerial part					
			Gastralgia		Aerial part					
			Headache		Aerial part		Decoction<br>or powder, or<br>dried aerial<br>part mixed<br>with honey			
			Ear diseases		Aerial part		Juice from<br>ground<br>fresh part<br>(drops to<br>be instilled)			
Coridothymus<br>*capitatus (*L.) Reichenb. Fil.<br>(= *Thymus c.* (L.) Hoff. et Link.*)*	Zaater	Timo	Antiseptic<br>Fight against tinea<br>Antibacterial,	Mouth wash<br>Expectorant,<br>balsamic,<br>digestive	Leaves<br>Leaves<br>Aerial part	Flowering<br>tops<br>leaves	Decoction<br>Poultice from<br>crushed	Infusion		7,9,16,17,18,26,28,29,<br>30,32
				specific against<br>*Ancylostoma*<br>*duodenalis*		Poultice<br>from<br>crushed<br>leaves	Decoction		In Italy<br>used<br>as spice on<br>meat and<br>food	
				Antiecchymotic		Leaves		Compresses<br>imbued with<br>decoction on<br>affected area		
			Cough		Leaves		Decoction			
			Headache		Leaves		Decoction			
			Antipyretic	.	Leaves		Decoction			
			**Vermifugue**	**Antihelmintic**	Leaves	**Leaves**	Decoction	Infusion		
			depurative<br>gastralgia		Leaves		Decoction			
			Diarrhoea and<br>vomiting of<br>newborn,		Aerial part		Decoction			
			makes the<br>delivery easier<br>Female sterility		Aerial part		Decoction			
			Sterility		Aerial part		Mixture with<br>oil and flour<br>(fasting)			
			Mild analgesic		Leaves		Decoction			
			Cardiotonic		Leaves		Decoction			

				.						
Lavandula stoechas L.	Halhal	Lavanda	Antiseptic in	Spasmolytic,	Leaves	Flowers	Decoction	Infusion		
			intestinal	mild sedative					In Italy	7,15,16,17,18,28,29, 41,48
			trouble	antihysteric					*L.officinalis*	
			gastralgia	antiseptic (e.u.)				Infusion	*L.* is also	
			Antihysteric	laryngitis	Flowers		Decoction	(by gargling)	used for the	
			Asthma	inflammations				Decoction	same purposes	
			**Antitussive**	**Antitussive**	Flowers		Fresh flowers to be eaten			
				Lumbago	Leaves		Powdered			
				Painful joint	Flowers		mixed with			
							olive oil	Rubbing of		
			Antalgic	Rheumatism			Essential oil	dried flowers		
Marrubium vulgare L.	Marroubia	Marrobio	**Hypotensive**	**Antihypertensive**				Infusion,		7,11,13,14,16,17,18, 26,27,28,30,33,37, 45,48
	morroubia,			Digestive,	Whole plant	Flowering	Decoction	tincture		
	omerroubia		Hypoglycemic	choleretic,	"	tops, leaves				
			Gastric diseases	expectorant,	"					
			**Cardiotonic**	**Cardiotonic**						
				antipyretic,	"			Cataplasm		
				antianemic,	"			from boiled		
			Pulmonary		"			aerial part		
			antiseptic		"		Decoction	Tincture or		
			Head cold		"		Sap inhalation	infusion from		
								powdered		
								flowers		
			**Depurative**	**Depurative**	Whole plant	Flowering tops	Decoction			
			Burns	of the blood	Whole plant					
			forunculosis	in jaundice,	Flowering tops	Leaves	Decoction			
			vulnerary	galactophore			Decoction			
			Decays	emmenagogue	Flowering tops		Decoction			
				Antimalarial	Whole plant		Decoction			
				Infection of feminine genital apparatus		Leaves		Decoction as washing solution		
						Leaves	as mouthwash	Decoction as washing solution		
			**Haemorrhoids**	**Haemorrhoids**	Whole plants		Powder (e.u.)			
			Ear diseases		Whole plants		Drops of			
			Abscesses				maceration			
			Warts		Flowers		in olive oil			
			**Rheumatism**	**Rheumatism** painful articulations		Whole plant		Cataplasm		
							Decoction	of boiled		
								aerial part		
			**Liver diseases**	**Liver protecting**	Leaves	Whole plant,	Decoction	Decoction		
			Urinary disorders			aerial part				
			Trachoma				Decoction			
							Powdered			
							cooked			
							with butter			
Mentha sp. pl.	see the	Menta	see the following	Anesthetic	see the following	Leaves and	see the following	Tincture		
	following		*Mentha spp.*	antiseptic	*Mentha spp.*	flowering	*Mentha spp.*	many uses		
	Mentha sp.div.			spasmolytic		tops			In Italy	7,16,17,18,27,28,29, 31,42,44
				digestive					many uses	
				refreshing				Infusion,	of *Mentha* are	
				liver protecting		Leaves and		tincture	generically	
				cholagogue		flowering			referred to	
				carminative		tops			different	
				balsamic					species	
				Sedative		Leaves and		Infusion,		
				Jaundice		flowering		tincture		
				Analgesic		tops				
				Fight against scabies		Leaves and		Infusion for		
				Insect bites		flowering		washing		
						tops		Rubbing		
Mentha pulegium L.	Flayyou	Puleggio	Headache	Stomachic	Aerial part	Flowering	Hydro-	Infusion		7,11,16,17,26,28,29, 34,48
			gastralgia	Digestive		plant	distillate			

				Neurotonic					
			Aerophagy	Neuralgia	"	Leaves	Essential oil	Locally rubbed	
			**Cold**	**Cold**			rubdown	Infusion with	
			**Antitussive**	**Antitussive**	"		Compresses	honey	
							of		
							essential oil		
							applied on		
							infant navel		
			Migraine		"		Essential oil		
				Mouth and throat		Leaves	massage on	Infusion as	
				inflammations			forehead	gargling	
			Fever, Flu		Whole plant		Decoction		
			respiratory disorder						
			gastralgia		"		Decoction		
			diarrhea		"				
			Asthma		Leaves		Fumigation		
Mentha spicata L.	Naanaa		Antipyretic	Neurotonic	Leaves	Leaves	Poultice	Maceration in	
			Rheumatism		"		Poultice	olive oil to rub	7,17,18,22,24,25,28, 29,48
			Warts		"		crushed and		
							locally		
							applied		
			Biliary disorder		Leaves		Decoction		
			belly pains				"		
			Flatulence				Decoction		
							in mixture		
							with fennel		
			**Gastralgia**	**Gastralgia**	Leaves	Leaves	Decoction	Infusion or	
				Anti-diarrheal			in mixture	decoction	
				Eupeptic			with *Verbena*		
			Menstrual pains				*officinalis* L.		
			Abortive				Decoction		
							in mixture		
							with *Thymus* sp.		
			Infant aerophagy		Leaves		Decoction		
							in mixture with		
							Arabic gum		
				Sedative for headache		Whole plant		To eat	
				and neuralgia					
				Antitussive,				Decoction	
				antiasthmatic					
				Insect bites		Leaves		Leaves rubbed	
								on affected area	
Mentha	Naanaa	Menta	Abscesses	Head ache	Leaves		Crushed		
suaveolens Ehrh.			furunculosis				leaves		7,9,18,24,25
							or decoction		
							(e.u.)		
			Fever		Leaves		Decoction		
							(to rubdown)		
			**Tooth ache**	**Toothache**	Leaves		Decoction		
							as		
							mouthwash		
Ocimum basilicum L.	Hbak	Basilico	Headache	Digestive,	Leaves,	Aerial part	Decoction	Fresh leaves	
			Dermatosis	Stomach ache	flowers		(e.u.)	used as spice	7,15,16,17,18,20,23, 25,27,29,37,41,42 44,45
				Intestinal trouble,					
				aromatic,		Aerial part			
				For insect bites,				Locally	
				Lenitive				applied	
				for the skin,				rubbing	
								on the skin	
				Mouth and throat		Leaves		Infusion as	
				inflammations				gargling or as	
				in gingivitis				mouth wash	
			Female sterility	Neurotonic	Flowers	Aerial part	Decoction	Infusion	
				in insomnia			for vaginal		

				and hysteria			wash			
				.						
Origanum majorana L.	Mardqouch	Maggiorana	**Antitussive**	**Antitussive,**	Leaves	Leaves and	Dried and	Infusion		7,16,17,18,20,23,24, 25,26,27,28,29,37, 44, 45,47,48
				Aromatizant,	Leaves	flowering	powdered +		Mainly used	
				digestive	Leaves and	tops	Butter + sugar		as spice	
				Antineuralgic	Flowers		(fasting)		on foods	
			cold				Essential oil	Infusion		
							(e.u.)			
			Mucolytic		Leaves		Decoction			
			Aperitive				Decoction			
			Gastralgia							
			Choleretic		Aerial part		Infusion			
			**Diuretic**	**Diuretic**	Aerial part		Infusion	Infusion		
			Dental				Fresh leaves			
			disorder				applied on			
							teeth			
			Menstrual pains		Leaves		Decoction			
				Lenitive,		Whole plant		Maceration in oil		
				antiecchymotic				locally applied		
				Headache		Flowering		Infusion		
						tops				
Rosmarinus	Klil,	Rosmarino	**Liver**	**Cholagogue**	Aerial part		Decoction	Infusion,	fresh leaves,	
officinalis L.	iklil		**protector**	**choleretic**				maceration	used as spice	7,11,13,15,16,17,18, 22,23,24,25,26,27, 28,29,32,33,34,36, 37,39,40,41,42,43, 45,47, 48
			laxative	antilithiasic						
				digestive,						
				eupeptic						
				Anti-diarrhoeic						
				Aromatizing,						
			**Cold, bechic**	**Antitussive**	Aerial part		Decoction			
			bronchitis	Antiseptic .	Aerial part		with or			
			**Asthma crisis**	**Antiasthmatic**		Flowering	without	Infusion		
						tops	*Thymus*			
				Fight against		Leaves and		Infusion		
				perspiration		flowers				
			Tonic	In promoting	Aerial part		Decoction	Infusion		
				cutaneous blood	Leaves		Maceration	as bath		
				circulation			to rub down			
				In regulating blood		Young stem		Infusion		
				pressure						
			**Rheumatism**	**Rheumatism**			Powdered	Infusion or		
							and mixed with	maceration		
							honey	in olive oil		
			Infants	Greasy skin	Leaves	Leaves	Powdered	Maceration		
			buttock	In case of			with honey	in wine		
			erythema	tiredness,			Powdered	as baths		
							(e.u.)			
				For coming ahead		Stem		Compresses		
				abscesses				imbued with		
								decoction		
						Leaves		Infusion		
				Cicatrizant, mild						
			Otitis	Mouth wash	Leaves		Maceration			
							locally			
							dropped			
			Antipyretic	To stop hair loss and Fight against	Aerial part	Leaves	Decoction for	Alcoholic extract mixed with root of		
				chilblains			rubdowning	*Arctium lappa*		
								and		
								leaves of		
								Urtica dioica		
				Stiffneck				Cataplasm		
				Antitinea				rubbed on		
								the scalp		
								mixed with		
								olive oil		
			Post-partum	Mild sedative for		Leaves		Infusion		
			troubles	CNS	Leaves		Hip bath			
			**Renal disorders**	**Diuretic**	Leaves	Leaves and	Powder mixed	Infusion		

						flowers	With honey	Infusion		
Teucrium capitatum L.	Gattabet lajrah	Canùtola	**Cicatrizant**	**Cicatrizant** Antiseptic,	Leaves	Flowering tops	Powder or poultice (e.u.)	Infusion to wash little		16,17,18,22,26,28,29
(= *T. polium* L.)			Cardiotonic		Leaves		Decoction	wounds		
				Antiscorbutic Antipyretic		Whole plant		Infusion Decoction		
**32 Lauraceae**										
Laurus nobilis L.	Rand	Alloro, Lauro	**Antirheumatic**	**Antirheumatic** Stomachic,	Fruits	Leaves	Decoction (e.u.) or	Oil from fruit Infusion	Leaves are used to aromatize foods	7,10,11,15,16,17,18, 20,21,22,23,24,25 26,27,28,29,39,41, 43,44,45, 47,48
				aromatic, carminative	Leaves	Fruit	oily extract locally applied			
			Sedative for restless sleep of infants	antiparasitic	Leaves Fruit	Leaves	Distilled water Decoction	Fresh leaves put into the rooms for repelling insects		
				Tonic in asthenia and weakness	Leaves	Leaves		Infusion or decoction		
			Anal fistula Jaundice	Antiseptic for minor wounds Mouth wash			Decoction (e.u.) Decoction with honey			
			**Expectorant**	**Expectorant** balsamic	Leaves	Leaves	Used as poultice in	Decoction		
			Dermatosis	Scalp dermatosis and dandruff	Fruits	Leaves, fruits	olive oil	Oil from fruit locally rubbed		
				Antipyretic		Leaves		Infusion		
				Sedative for abdominal and menstrual pains				Decoction with *Matricaria chamomilla*		
				Excessive perspiration of feet		Leaves		Decoction as foot bath		
				Haemorrhoids		Fruits		Maceration in olive oil e.u.		
**33 Leguminosae**										
Glycyrrhiza glabra L.	Erq Essous, Arab essous, Oud essous	Liquerizia	Asthma, **Respiratory affections**	**Antitussive, Expectorant** Emollient, Vulnerary cicatrizant	Roots	Roots	Infusion	Decoction		7,9,11,17,18,26,27 29,30,33,35,41,43 45
			Gastric ulcers (cortisone like)	Sedative for stomach-ache Antiprostatic adenoma Mouth and throat inflammations	Roots		Infusion	Infusion mixing *Malva sylvestris*, *Foeniculum vulgare* var. *piperitum*		
Lupinus sp. pl. In Tunisia only *L. micranthus* L., L.hirsutus, L. luteus	Termouss	Lupino	Used only as fodder	Hypoglycemic, Antihelmintic. Fight against skin parasites	seeds	Seeds	Not specified	Decoction, tincture, extract	Seeds must be warmed before use to deprive them from toxicity	7,22,24,26,28,29,35 40, 48
				Cosmetic (face mask)		Flour from seeds mixed with water				
Medicago sativa L.	Fassa	Erba medica	**Reconstituent** recalcificant antianemic	**Reconstituent** Antiscorbutic, Eye inflammations		Leaves		Mainly used as fodder		26,27,28,29
			Antihemorrhagic			Fresh leaves		Cataplasm or eye wash		
Trigonella foenum- graecum L.	Helba	Fieno greco	**Reconstituent**	**Reconstituent** aperitive,	Seeds	Seeds	Powdered seeds	Decoction from		9,12,16,17,18,26,28, 29

			Antidiarrheal Intestinal colic fever	Dietetic, nutrient,	Seeds		mixed with water and sugar or	powdered seeds		
			Children gastralgia	Expectorant		Seeds	decoction of roasted seeds	Cataplasm from powdered seeds locally applied		
			Bechic		Seeds		Powder of roasted seeds mixed with olive oil			
			cold	In case of nephritis (lenitive)		Dried seeds		Decoction mixed with *Avena* seeds		
			Angina (sore throat)				Decoction for gargling			
			Abscesses, furunculosis		Seeds		Powdered seeds mixed with water and locally applied			
**34 Liliaceae**										
Allium cepa L.	Bsal	Cipolla	Hemiplegia	Diuretic	Bulb	Bulb	Decoction mixed with honey and chick pea	To eaten fresh or boiled	Use reported only in Agri Valley (Lucania)	7,9,10,11,15,16,17,18, 20,21,22,24,25,27,29, 39,40,41,42,44,45,47, 48
				Liver protector		Bulb		Maceration in "Marsala" wine		
			**Hypoglycemic**	**Hypoglycemic**			Decoction mixed with honey	To eaten fresh or boiled		
			**In making abscesses come to ahead**	**In making abscesses come to ahead**	Bulb	Bulb	Poultice locally applied	Cataplasm of squashed bud		
				Sun burns						
			Antiseptic	Hemostatic	Bulb	Bulb	Poultice	"		
			Sunstroke	Cicatrizant			applied on head	"		
				Hoarseness		Bulb		To be eaten row		
				Otitis				Ointment with locally applied		
				Emollient for intestinal tract		Bulb		Tincture in wine		
				Fight against rhagades				Decoction		
				For excessive sweating of feet				Decoction as Foot bath		
				Hair loss				Ointment from Boiled bulb		
				Fight against drunkeness		Bulb		Maceration in milk		
Allium sativum L.	Thoum	Aglio	**Hypotensive**	**Hypotensive**	Cloves of bulb	Cloves of bulb		Fresh bulb cloves to eat		7,11,15,17,18,20,21, 22,23,24,25,27,29,39 40,41,44, 45,47,48
				Antibacterial, antiviral for respiratory and intestinal tract	Cloves of bulb					
				Antiatherosclerosis						
			Dizziness, Loss of consciousness	Hypoglycemic	Cloves of bulb		Mixture with vinegar and lemon			
			**Cardiac analeptic**	**Cardiotonic**	Cloves of bulb		Not specified			
			**Haemorrhoids**	**Haemorrhoids**			Not specified			
				. Tooth ache		Bulb		Squashed bulb Locally applied		
			Antivenom for insect or scorpion stings,	Stiffneck	Cloves of bulb	Whole plant	Mixed with vinegar and bran	Cataplasm from the plant boiled in milk		
			Hemostatic	Warts and corns				Juice from cloves		
			Dental decay		Cloves		Burned and grounded with pepper and honey	or squashed cloves with olive oil locally applied		

			Alopecia		Cloves		To rub on the scalp			
			**Vermifuge**	**Antihelmintic**<br>Cholagogue<br>Antiarthritic.<br>antirheumatic		Cloves		Fresh bulb cloves to be eaten<br>Maceration in petroleum, filtered through a linen then smeared on painful joints		
Asparagus officinalis L.	Sakkoum	Asparago	**Diuretic**	**Diuretic**<br>haemorhoids	Rhizome	Rhizome<br>Shoots	Decoction	Decoction<br>Decoction as wash		7,13,20,21,26,27, 29, 45, 48
In Italy<br>*Asphodelus albus* Miller	Absent in Tunisia,	Asfodelo<br>Porraccio		Fight against alopecia	Tuber	Tuber		Decoction as shampoo	Toxic plant	7,15,26,
In Tunisia and in Italy<br>*A. microcarpus* Salzm et Viv.(=*A. aestivus* Brot.= *A. infestus* Parl.)	Berouag	Asfodelo<br>Porraccio	Earache	Warts and corns	Tuber	Tuber	filled with olive oil for local instillations	Cataplasm of squashed tuber		7,11,14,17,18,28, 35,39,43
				Antirheumatic		Root		Cataplasm from soaked root with olive oil Applied on painful joints		
				Fight against catarrh		Tuber		Decoction		
				Cicatrizant				Mashed tuber for rubbing		
Urginea maritima (L.) Baker	Aansal	Cipolla marina	Helps children with back-wardness to walk	Cardiotonic<br>Diuretic	Bulb	Bulb	Maceration in oil and local rubbing	Infusion of powdered bulb		7,13,16,17,18,26,28, 29
				Abscesses				Sliced bulb locally applied		
			Earache		Bulb		Oil from cooked bulb for auricular instillation			
**35 Linaceae**										
Linum usitatissimum L.	Kettan	Lino	**Antitussive**	**Expectorant**	Seeds	Seeds	Poultice of ground and powdered seeds (the same mixed to mustard applied on Thorax)	Cataplasm of boiled powdered seeds	The oil from seeds is also used for oil-paint	7,13,15,16,17,18,20, 23,25,26,27,28,29 32,40, 44,45,47,48
				Lenitive for the skin<br>For itching<br>Furuncle				Decoction or cataplasm locally applied		
				Lenitive for intestinal tract		Seeds mucilage		Maceration in water		
				Lenitive for haemorrhoids		Seeds		Oil from the seeds to rub locally		
			Asthma<br>To get easy delivery		Seeds		Chewed and swallowed			
			Measles (before the eruption)				Decoction to drink			
			**Constipation** gastritis	**Laxative**	Seeds	Seeds		Crushed seeds in water		
			Impetigo<br>to make gums abscesses to a head		Seeds		Ground seeds mixed with water, locally applied			

				Lenitive in phlebitis		Seeds		Compresses		
								soaked in		
								decoction		
**36 Malvaceae**										
Malva sylvestris L.	Khobbiza	Malva	Cicatrizant	Emollient ,	Leaves	Leaves	Ground leaves	Infusion as		7,9,10,11,12,13,14,15
			bee or insect	lenitive for		Flowers	locally applied	washing or		16,17,18,20,22,23
			stings and bites	the skin				cataplasm from		24,25,26,27,28,29,31
				Aphtae				squashed leaves		33,35,37,39,40,41,43
				Mild laxative				Infusion		44,45,47,48
			Furuncles	soothing mouth and throat	Leaves and		Fruits mace-			
				inflammations	fruit		ration in	Infusion as		
							olive oil	gargling		
			Eyes inflammation	Intestinal and urinary	Leaves		Decoction	Infusion		
				inflammation and			instilled into			
				flatulence			eyes (eye salve)			
			Asthma,	Cough	Aerial parts		Decoction	Infusion		
			**Cold**	**Cold**			to drink			
			Anuria,							
			Kidney stones							
			Toothache,		Fruits		Decoction			
			gingivitis				as mouthwash			
				Dislocation (sprains)		Leaves		Boiled leaves		
								Locally applied		
**37 Moraceae**										
Ficus carica L.	Karma	Fico	Haemorrhoids	Laxative,	Dried fruits	Fruits	Fruits to eaten	Infusion or		7,9,11,15,16,17,18,20
	(fruit :			anti-inflammatory				decoction of fruits		24,25,26,27,28,29,30
	karmous)		Gastralgia	Antitussive				boiled in water		31,34,39, 44, 45, 47, 48
					Dried fruits			or in milk		
			**Warts and**	**Warts and**	Latex	Latex	locally	Locally		
			**corns**	**corns**			applied	applied		
				Insect sting		Latex				
			Soothing		Leaves		Poultice of			
			respiratory				leaves			
			diseases				macerated in			
							olive oil			
			Eye leukoma		Leaves		Ground	Split figs		
			**In gums**	**In gums**		Fruits	leaves	boiled in		
			**abscesses**	**abscesses**			locally	tepid		
			to ahead	Aphtae			applied	water and		
							impregnated	locally		
							in olive oil	applied		
				Lenitive for		Leaves		Crushed leaves		
				haemorrhoids				locally applied		
Morus alba L.	Tout	Gelso bianco	Hemorrhoids	Expectorant,	Leaves	Fruits	Decoction	Syrup from		7,16,17,18,23,24,26,
	abiadh			Laxative ,			to drink	squashed fruits		27,29
				astringent,	Leaves					
			**Diuretic**	**Diuretic**	Leaves	Leaves	Decoction	Infusion		
							to drink			
				Hypoglycemic				Decoction		
			Eczema	Antihelmintic	Latex	Bark	Locally	Decoction		
							applied			
Morus nigra L.	Tout akhal	Gelso	Toothache	see *M. alba*						see *M. alba*
**38 Myrtaceae**										
Eucalyptus sp. pl.	Kalatous,	Eucalipto	**Antitussive**	**Balsamic,**	Leaves	Leaves	Leaves	Decoction		7,9,11,15,16,17,18,20
	Safsaf			**antiseptic of**			decoction			22,23,24,26,27,29,39
				**respiratory**			impregnated			44,47, 48
				**tract**			in olive oil			
				Antiparasitic			(e. u.)			
			Chilblain		Leaves	Bark	Compresses	Decoction	The scent	
			**Antipyretic**	**Antipyretic**			imbued with		of fresh	
				Hypotensive			decoction		leaves	
							(e.u.)		repels	
			Decayed		Leaves		Decoction as		insect	

			tooth				mouthwash			
				Hypoglyemic		Leaves, flowers		Decoction	Only in Campania region	
Myrtus communis L.	Rihan	Mirto, Mortella	for soothing ulcers and gastralgia	**Balsamic, antiseptic of respiratory tract**	Fresh fruits	Leaves	To be eaten or drunk as decoction	Infusion	To use very cautiously because it may cause poisoning In Sardinia (Italy), the fruits are employed in preparing the home made	7,11,16,17,18,25,26, 27,28,29,39,41, 44,45 48
			Acute diarrhea	Diuretic	Flower		Decoction	Infusion		
			**Cough and rhinitis**	Cicatrizant	Fruits		Decoction			
				Astringent and anti-hydrotic for feet and armpit		Leaves		Toasted and powdered leaves are applied locally		
			Gingivitis				Decoction as mouthwash			
			Rheumatic pains		Fruit oil		Locally applied			
				Arthritic pains		Stems		Decoction to drink		
**39 Oleaceae**										
Olea europaea L.	Zitouna, Chojret zitoun	Olivo	**Constipation** Jaundice	**Mild laxative** cholagogue, choleretic	Oil	Fruits	Oil from the fruit to drink	Oil from the fruit to drink		7,9,10,11,12,13,14,15 16,17,18,20,21,22,24 25,26,27,28,29,30,33 34,39,40,41,42,43,44, 45,47,48
				Anti-atherosclerosis Emollient for the skin Fight against burns	Oil		Olive oil added to lemon juice to drink			
			Treatment of tubercolosis	Vitaminic	Oil		Olive oil mixed with egg and orally taken			
			Flu, in soothing of chilblains	Fight against gout For hair strengthening	Oil	Leaves Oil	To rub over all the body	Decoction Oil locally applied		
			**Hypotensive**	**Hypotensive**	Leaves	Leaves	Decoction	Decoction		
			**Otitis**	**Otitis**	Leaves	oil	Imbued leaves in warm olive oil and mixed with salt instilled into ear	Drops of oil into the ear (auricular drops)		
			Trachoma		Leaves		Leaves sap as ocular drops			
			Toothache		Leaves		To be chewed			
			Chapped lips mouth ulcers gingivitis				Decoction as mouthwash			
			tongue inflammation (glossitis)	Antipyretic	Leaves		Decoction			
**40 Papaveraceae**										
Papaver rhoeas L.	Bougar'oun	Papavero, Rosolaccio	Epistaxis **Tranquillizer**	**Mild sedative**	Whole plant	Petals	Powder (e. u.)	Infusion	Seeds are used in pastry	7,9,10,11,14,16,17,18 20,21,23,24,25,26, 27,28,29,30,31,32,33 37,39, 43, 45,48
			**Antitussive**	**Antitussive** Against gastralgia	Whole plant		Decoction			
				Eye salve for Eyes and eyelids inflammation Toothaches		Petals		Infusion		
**41 Pinaceae**										
Pinus pinaster Ait.	Snouber	Pino marittimo	**Antitussive**	**Balsamic for respiratory tract**	Leaves Leaves	Buds	Decoction (e.u.)	Decoction		7,16,22,26,28,29, 40,47,48
				Antiseptic		Resin		As cataplasm		
			Antiulcerous Cicatrizant	Cicatrizant for minor wounds		Resin		Warmed resin locally		

				Painful articulations				applied	
**42 Polygonaceae**									
Polygonum aviculare L.	Gordhab	Corrigiola, Centinodia	In soothing of gastralgia, intestinal diseases	Astringent, cicatrizant	Whole plant	Aerial part	Decoction	Infusion	7,16,17,18,26,27,28 29,30,33,35,40,45, 48
			diarrhea<br>Asthma	Renal trouble	Whole<br>plant				
			**Renal trouble**	**Diuretic**		Aerial part		Infusion	
In Tunisia									
Rumex tuberosus L.	Hommidha, Hommidh	Absent in Italy	Carminative<br>Asthma, antitussive	-	Whole plant	-	To be eaten fresh	-	17
			jaundice		roots		Aqueous macerate		
R. aristidis Coss	Hommidha	Absent in Italy	aerophagy		Aerial part		Internal use		16
R. crispus	Snanoun		astringent		roots		Not specified		28,29
In Italy									
R. acetosa L. and *R. acetosella* L.		Acetosa<br>Acetosa minore	Jaundice	Cholagogue	Roots		Maceration		
				Antiscorbutic<br>Diuretic<br>Mild laxative<br>Depurative		Leaves and stems		To be eaten fresh<br>Infusion	7,27,39,48
				Anti arthritic<br>Remineralizant		Young plant		Infusion	
						Leaves		Infusion	
**43 Portulacaceae**									
Portulaca oleracea L.	Blibcha, Bendlaqa	Porcellana, Porcacchia	Flatulence	Emollient<br>Diuretic<br>Antiscorbutic	Whole plant	Leaves and juice	Fresh poultice applied on belly	To be eaten raw as salad	7,17,18,23,24,26,28, 41
				Anti-inflammatory of intestinal tract		Leaves			
				Anti-pyretic		Juice from fresh plant		To assume	
				Antihelmintic		Whole plant		Infusion	
				Gums inflammation		Aerial part		Infusion as gargling	
**44 Primulaceae**									
Samolus valerandi L.	Oudhina	Not specified	Otitis	Antiscorbutic<br>Gastralgia<br>Cholagogue	leaves	Whole plant	Ground and added to olive oil Warmed, then drops instilled into ear	The ash from the fresh plant melted in water	7,16,17,26
				Vulnerary				The ash locally applied	
**45 Punicaceae**									
Punica granatum L.	Romman	Melograno	Gastric ulcers	Antihelmintic, Thirst quenching (seeds)	Fruit bark	Bark of the root, stems, peel, seeds	Decoction from dried bark	Decoction	7,17,18,20,24,25 26,27,28,30,32,40, 45,48
			**Gingivitis**	**Gingivitis**<br>pyorrhea	Fruit bark	Bark of the root, stems,	decoction as mouthwash	Decoction	
				Antitussive		Juice of ripe fruits		Decoction	
				Hemostatic		Bark			
				Antipyretic		Juice of unripe fruits			
			Diarrhoea	Diarrhoea	Fruit bark	Fruit peel	Decoction from dried bark	Decoction	
			Hypoglycemic<br>Hypotensive		Fruit		Decoction		
				Arthritic pains		Fruits		Decoction	
**46 Rafflesiaceae**									
Cytinus hypocistis (L.) L.	Daghmous	Mugghi-gnero	Auricular diseases	Astringent hemostatic	Leaves	Juice of fresh plant	Exudate from warmed		12,17,26,28

						locally applied	fresh leaves as ear drops			
**47 Ranunculaceae**										
Clematis flammula L. and *Clematis vitalba* L. .	Nar barda	Fiammola<br>Vitalba	**In soothing of rheumatic pains**	**Revulsive in rheumatic pains** (squashed fresh young stem locally applied can cause skin ulcers)	Aerial parts	Leaves	crushed and applied not directly on painful joint	Infusion	Only external use. Young leaves and buds are smoked as tobacco substitute Young stems can be boiled omelettes	7,14,15,16,17,18,20, 26,27, 45, 47,48
				vein ulcers and furuncles		Leaves		As wash for e.u.		
				Antiodontalgic		Stems				
				Headache		Aerial parts		Compresses imbued with decoction (e.u.)		
Nigella damascena L.	Sinouj, Habba souda	Fanciullaccia	Cardiac analeptic Mucolytic Rheumatism Gastric ulcer	Diuretic Carminative Emmenagogue	Seeds	Seeds	Powdered seeds mixed with honey (internal use)	Infusion	In Italy it is less used than the *N. sativa* L.	7,14,16,17,28,29
			Trachoma		Seeds		Powdered seeds mixed with honey			
			Antihelmintic		Seeds		Decoction			
			Toothache		Seeds		Powdered seeds mixed with vinegar or decoction as mouth wash			
			Headache Sinusitis		Seeds		fumigation			
In Italy: *Ranunculus bulbosus* L. and *R.b* subsp. *bulbosus*	Absent in Tunisia	Ranuncolo		Revulsive in rheumatic and arthritic pains (e.u.) can cause skin ulcers	Whole plant	Green parts	Decoction (e.u.)	Only external use	Toxic plant Other species of *Ranunculus* are also used in Italy	7,14,26,45
In Tunisia : *R.. macrophyllus*	Kaf jrana	Absent in Italy	Dermatosis	-	Leaves	-	Not specified	-		16,17,18
			Bronchitis		Whole plant		Poultice (e.u)			
**48 Resedaceae**										
Reseda alba L. (= *R. suffruticulosa L.)*	Baasous khrouf	Reseda	**Eye inflammation** Ocular leukoma	**Eye inflammation** Lenitive for the skin	Leaves	Leaves	Drops of the decoction as eye salve or chewed leaves locally applied or chewed and squeezed leaves instilled in drops	Compresses Imbued into decoction Leaves locally applied	Mainly used in Italy in perfumery	17,28,45
Reseda luteola L.	Baasous khrouf	Guaderella, Biondella	Children's diarrhea colic Fight against poisoning	Diaphoretic Diuretic	Leaves	Flowering tops	Infusion	Infusion		7,18,26,28
**49 Rhamnaceae**										
Rhamnus alaternus L.	Oud el Khir	Linterno	**Jaundice**	**Jaundice** (use known only in Sardinia region)	Aerial parts	Young stems	Decoction	Fumigation to inspire	For its strong action it must be associated	7,12,15,17,18,26 28,29
				Drastic		Fruits		Infusion		

				purgative					with *Malva sylvestris* and . *Althaea officinalis*	
**50 Rosaceae**										
Prunus persica (L.) Batsch	Khoukh	Pesco	Otitis	Vitaminic	Fresh leaves	Fruits	Decoction or sap from squeezed fruit	To be eaten	Mainly alimentary use in Italy	7,16,17,27,29,41,48
				Antitussive		Flowers		Decoction		
			Hypotensive Diuretic		Leaves		Decoction	mixed with milk		
			**Purgative for children**	**Laxative**		Fruits	Decoction as syrup	To be eaten		
Rosa canina L.	Nesri	Rosa di macchia	Fight against asthenia Cardiac analeptic	Astringent, tonic and lenitive for the skin	Flowers	Petals still in buds	Distilled water (with or without egg)	Maceration	In the past hips were collected as source of vitamin C	7,10,16,17,18,23,26, 27,28,29,41,42, 45,48
				Antitussive Antiscorbutic		Flowers		Infusion or decoction		
				Hemorrhoids Bladder stones		Leaves		Infusion		
Rosa gallica L.	Ward	Rosa rossa	Ocular diseases	Antiscorbutic Tonic Laxative	Flowers	Cynorrhods (hips)	Distilled water as eye salve or imbued compresses locally applied		They are still used in homemade syrups, jams	7,17,25,26,27,28,29 44
			Purgative		Petals		Decoction			
			To make abscesses come ahead		Petals		Petals boiled in salted water and mixed with olive oil			
Rubus ulmifolius Schott.	Alliq, Tout el alliq	Rovo	Headache Burns	Astringent, antidiarrhoeic lenitive for the skin	Leaves	Leaves, fruits	Poultice from ground leaves	Infusion		7,9,10,11,14,15,17,18 20,22,25,26,28,33,34 35,37,40,42, 44,45, 47,48
			Gingivitis aphtae angina	Hypotensive		Leaves	Decoction as mouthwash or gargling	Decoction		
				Antiscorbutic Diuretic In nephritis and prostatitis		Roots Leaves		Decoction		
				Mouth wash Thirst quenching		Fruits		Decoction		
				Hypoglycemic Antitussive Throat inflammations		Roots				
				Bronchial catarrh		Bark		Decoction		
**51 Rutaceae**										
Ruta graveolens L. *R. angustifolia* Pers. and *R. chalepensis* L.	Fijel	Ruta	Children gastralgia profuse diarrhoea with vomit	Antiparasitic Abortive Protecting blood vessel , antiecchymotic	Leaves	Leaves	Decoction	Infusion, maceration (rarely used)	Toxic plant The leaves are used to aromatize spirits	7,9,12,13,15,16,17,18 20,22,23,24,25,26,27 28,29,30,32,34,39,40 41,42, 43,45,47,48
						Aerial part		Compresses imbued with decoction	Stems and leaves are put into rooms infested by mice and flees to repel them	
				Toothache		Stem		Locally applied		
				Digestive		Whole plant		tincture or medicated wine		
						Fresh plant		Maceration in water		
			**Hypotensive**	**Hypotensive**		Fresh plant		Infusion		
			**Emmenagogue**	**Emmenagogue**		Leaves		Infusion		

			**Antihelmintic**	**Antihelmintic**		Leaves				
				Antimalarial		Root		Decoction		
			Fight against infants aerophagia	(use known only In Sardinian region)	Leaves		Eaten by feeding mother			
			Antitussive		Leaves					
			**Headache** Rhinitis	**Headache**	Leaves	Fresh plant	Poultice from squashed leaves locally applied	Distilled in water		
				Eye trouble		Leaves		leaves are chewed and the breath is spayed into the eye		
				Cicatrizant		Fresh plant		Juice on wounds		
				Urinary troubles , gall stones				Decoction		
				Abscesses		Leaves		Crushed with Olive oil e.u.		
Ruta montana (L.) L.	Fijel el jebel	Ruta finocchina	Rheumatism	Emmenagogue abortive Hypertensive	Roots or leaves	Leaves	Maceration in olive oil to rub	Infusion		17,18,26,28,29
				Antalgic antiinflammatory		Leaves	on painful joints	External use		
			Otitis and ear pain		Leaves		Drops from maceration in olive oil to rub or instilled into ear			
**52 Scrophulariaceae**										
Verbascum sinuatum L.	Saleh landhar, Mosleh landhar	Verbasco, Tasso barbasso	Ocular diseases	Psoriasis	Flowers	Aerial part	The sap from triturated and squeezed flowers	Decoction evaporated the filtered cataplasm	About this peculiar use known only in Sicily see in the text	10,16,17,18,20,24, 26,27,28,29,30,31 35, ,45,47, 48
				Hemorrhoids						
				To wash infected wounds			then filtered Used as eye salve drops	of crushed leaves		
				Lenitive for the skin				Leaves	In Sardinia	
				Antiasthmatic		Dried leaves		Infusion	leaves are ground with olive or castor oil, or boiled	
**53 Solanaceae**										
Capsicum annuum L.	Felfel	Peperoncino	Otitis	For digestion stimulation Antiscorbutic	Fruit (red)	Raw fruits	Maceration in olive oil as ear drops	To be eaten		7,9,16,17,18,20,24, 25,29,41,42, 44, 45,47,48
			Headache	Hemorrhoids Alopecia	Fruit		Dried powder soaked in olive oil	Tincture Cataplasm from fresh or	mixed with food to give flavour	
			**Rheumatism**	**Rheumatism**	Fruit		Maceration in water (e.u.)	dried crushed fruit mixed with olive		
			Cattle and sheep trachoma		Fruit		Dried powder applied in eyes	oil		
				Hypotensive		Fruits		To be eaten as condiment		
				Chilblains				Boiled fruits Locally applied		
				Dandruff and hair loss		Fruits		Tincture		
Lycium europaeum L.	Aoussej,	Licio,	Weeping	Antispasmodic	Fresh	Leaves	Decoction	Infusion		7,9,11,17,18,26,28,29

	Sakkoum	Spina- christi	eczema	Antihydrotic	leaves	collected before flowering	(e.u.)		
				Boils Foot pains		Leaves		Squashed leaves Decoction as Foot bath	
Lycopersicum esculentum Mill.	Tmatem	Pomodoro	Hypotensive	Vitaminc	Leaves	Fresh fruits	Decoction	To be eaten	9,11,17,23,24,27,40 45,48
			Furuncle	Antiarthritic Laxative	Fruit	Leaves	Decoction locally applied	Infusion	
				Insect bites (wasp and bees)		Fruits		Squashed to apply locally	
				Renal stones		Fresh leaves		To be drunk	
Solanum nigrum L.	Tmatem kleb, Tmitma Aneb edhib,	Erba morella	Eczema, erysipelas	Hemocathartic In painful joints and sciatic nerve diseases	Fruit	The whole plant	Sap from the squashed fruit locally applied		7,9,16,17,18,25,26 27,28,29,37,39,48
			Burns	Fight against itching due to hemorrhoids	Whole plant	Fresh leaves	Poultice	Poultice locally applied	
				Tooth ache		Fruit juice		Fumigations with A burning gauze Previously soaked with the juice of the fruits	
				Sedative of trouble of Respiratory tract		Whole plant		Infusion	
**54 Tamaricaceae**									
Tamarix gallica Poir.	Terfa	Tamerice	Conjunctivitis	Astringent Diaphoretic Diuretic	Whole plant	Bark Leaves	Fumigation of burned plant	Decoction Decoction	16,17,26,28,45
			In gastralgia soothing	Laxative Antipyretic	Leaves		Decoction		
			Bee stings		Leaves		Maceration in water		
			Tonic, fight against diarrhoea		Stem bark		Decoction		
**55 Thymeleaceae**									
Daphne gnidiun L.	Zez	Erba corsa	Jaundice	Hemocathartic Diaphoretic Revulsive, vesicatory	Stem bark	Bark before flowering	Fumigations	Decoction Locally applied To be used cautiously given its toxicity	7,13,14,15,16,17,18, 26,28,29,45
				Rheumatic pains		Bark		Poultice (e.u.)	
**56 Umbelliferae**									
Ammi visnaga (L.) Lam.	Nounkha, Noukha khella	Visnaga	**Diuretic,** Nephro- lithiasis Typhoid fever	**Diuretic** dilating coronary vessel, spasmolytic	Whole plant Whole plant	Ripe fruits	Decoction	Infusion, Extract, powder	16,17,18,26,28,29
Anethum graveolens L.	Chebt	Aneto	Diuretic, lithotriptic **Carminative,** stomachic Galactogenous	Aperitive **Carminative**	Fruit	Fruits	Decoction	Infusion	16,17,18,26,28,29
Anthriscus sylvestris (L.) Hoffm.	Kochbor	Cerfoglio selvatico	toxic	Diuretic	Not used	Aerial part		Abandoned for its toxicity	16,17,26,28,29

Apium graveolens L.	Klafes	Sedano	Chilblains	Aperitive	Aerial part	Fruits and	As footbath	Infusion		7,9,17,18,20,21,23,
				carminative		leaves				26,27,28,29,32,35,
			Measles	diuretic,	Aerial part	Aerial part	Ointment	Infusion		36,37,40,41,45,48
				uterotonic			mixed with			
				Antiscorbutic,		Leaves	goat grease			
				expectorant		Leaves		Decoction		
				Antihelmintic		Roots				
				Lenitive for sores		Fresh minced		Locally applied		
				and bruises		Leaves				
Carum carvi L.	Karwiyya	Cumino dei	Antispasmodic	Antiseptic	Fruits	Fruits	Decoction	Infusion		16,17,18,26,28,29
		prati	carminative	for the skin						
			heart analeptic	For stimulation of	Fruits	Fruits				
			Rheumatism	peripheral	Fruits			Infusion		
			galactogenous	blood circulation						
			**Gastralgia**	Uterotonic	Fruits	Fruits				
			Infants enuresis	Locally applied						
				promotes						
				milk secretion						
			Antitussive	**For stimulation**	Fruits	Fruits	Decoction			
				**of gastric**						
				**secretion**						
Coriandrum	Tabel	Coriandolo	Gastralgia	Digestive	Fruits	Fruits	Powder	Tincture,		7,17,18,26,27,28,29
sativum L.				**Carminative**				Infusion		
			**Areophagy**	aromatizant	Fruits	Raw fruits	Decoction	Powder		
				for foods						
									In	7,16,17,18,26,28,29
Cuminum cyminum L.	Kammoun	Cumino	Intestinal colic	**Carminative**	Fruits	Ripe fruits	Decoction	Decoction	preparing	
			gastralgia,	Digestive					liqueurs	
				diuretic						
				bitter tonic						
			Infant's	Infant's catarrh	unripe fruits	Fruit		Fumigations		
			**areophagy**				To be eaten	with fruits		
			Contraceptive		unripe fruits		To be eaten	mixed with		
			Abortive				mixed to	honey		
							Juniperus			
							communis			
							pseudofruit			
Daucus carota L.	Sfennaria	Carota	Chilblains	Source of	Leaves	Root	Decoction	Eaten raw or		7,10,11,13,16,17,18,
				provitamine A	Root		as footbath	as vegetable		23,24,25,26,27,28,29, 37,39,41, 43,47,48
			Orchitis							
			Aphrodisiac	Antidiarrheal	Seeds	Root	To be eaten			
				Carminative		Seeds		Infusion		
				Expectorant, cough		Juice from		Juice to assume		
				Bronchitis		roots		mixed with honey		
				Loss of voice		Leaves		Infusion of		
								Powdered leaves		
				Lenitive for		Root pulp		Poultice from		
				burns and sores				grated pulp		
				Furuncles		Root		Ointment from		
								squashed root		
								locally applied		
								to come ahead		
Foeniculum vulgare	Besbes	Finocchio	Infant's	Carminative,	Fruits	Fruits	Decoction	Infusion		7,9,11,16,17,18,20,21
ssp .*vulgare* . Miller			areophagy	digestive		leaves				22,23,24,25,26,27,28,
			**gastralgia**	**gastric acidity**		Fruits		To be eaten		33,34,37,39,41, 43,44
			antidiarrheal,							47,48
				Fight against						
and *F.v.* ssp. *piperitum*		Finocchio		hiccup		Roots		Decoction		
(Ucria) Couthino		selvatico		Antihelmintic						
			Dysuria	Expectorant	Fruits	Root	Decoction	Infusion		
			galactogenous	Diuretic		Leaves		Decoction		
				Antirheumatic						
				Emmenagogue						
				Fight against hydropsy		Leaves		Distillate from		
								infusion		
				Swollen eyes		Fruit		Compresses		
								imbued with		

								infusion		
Petroselinum	Maadnous	Prezzemolo	**Diuretic**,	**Diuretic**	Leaves	Fresh	decoction			16,17,18,22,23,25,26
crispum (Mill.) Fuss.			dysuria	Source of		leaves			Abandoned	27,28,29,37,40,41,
			renal stones	vitamine C		Fruits		Illegal use	in Italy for	44,45,47, 48
			nephritic colic	emmenagogue		Whole plant			its toxicity	
				abortive				Squashed and		
				Antiecchymotic		Root		mixed with		
								vinegar		
				**For stopping**						
			**Stops onset**	**milk**	Leaves	Leaves	Crushed	Cataplasm		
			**of lactation**	**increasing**			leaves			
			**Insect bites**	**Insect bites**	Leaves	Leaves or	rubbed on	Rubbed on the		
						juice	breast	skin		
				Antiecchymotic		Aerial part		Decoction in		
								vinegar		
				Eye inflammations		Juice from	Fresh leaves	Instillations		
						the fresh	locally			
			Gonorrhea		Leaves	plant	rubbed			
							Decoction			
							mixed to			
							cherry stem			
							(« queue de			
							Cerise »)			
							and maize			
							stigma			
				Antirheumatic		Leaves		Maceration in		
				Mild laxative for		Flowering		Crushed in olive		
				children		tops		oil		
				Headache		Fresh leaves		Squashed and		
								Locally applied		
Pimpinella anisum L	Habbet	Anice	Antipyretic	Digestive	Fruits	Fruits	Decoction	In preparing		7,16,17,18,23,25,26
.	hlawa		**Gastralgia**	**Antispasmodic**			Decoction	liqueurs		29,41, 44,48
				Carminative						
			**Galactogenous**	**For increasing**						
				**milk secretion**		Inflorescence		Decoction		
				Antiasthmatic		Fruits		Infusion		
Thapsia garganica L.	Deryes,	Tapsia	**Revulsive**	**Revulsive in**	Aerial part	Roots	warmed	Only e.u.		7,16,17,18,26,28,29
	bou nefaa		**in rheumatic**	**lumbago and**			leaves mixed	can cause		
			**pains**	**rheumatic pains**			with oil	severe rash		
			Abscesses				Locally			
							applied			
**57 Urticaceae**										
Urtica sp. pl.	Horriqa	Urtica	**Anuria**,	**Diuretic**,	Whole plant	Leaves	Decoction	Infusion		7,9,10,11,13,17,18,20
			enuresis	soothing for						21,22,23,24,25,26,27
			nephrolithiasis	the intestinal tract						28,29,33,35,37,39,40
			Chilblain,	Antigout						41,42,44,45,47, 48
				antirheumatic		Leaves				
				Haemostatic						
				Hemorrhoids				Infusion		
				Gallstones		Leaves		Decoction		
				Source of Fe						
				Blood depurative						
				For hair strengthening						
				Fight against dandruff				Infusion for		
				Cutaneous rash, eczema				shampoing		
				Antigout		Whole plant		Decoction		
				Menstrual pains						
**58 Verbenaceae**										
Verbena officinalis L.	Tronjia	Verbena	Cold	Diuretic	Leaves	Flowering	Decoction,	Infusion		7,16,17,18,20,24,25,
			Headache	bitter tonic		plant	infusion			26,27,28,29,30,33,40
			Antipyretic	Anti-diarrheal						42,44,45,48
			**Intestinal colic**	**Intestinal colic**		Dried leaves		Decoction		
			Gastralgia							
			Tranquillizer							
				Emmenagogue						
				Anti-rheumatic		Fresh plant		Mildly warmed		
				For lumbago and		Leaves		Crushed and		
				neuralgia				locally applied		

				Cicatrizant		Whole plant		Decoction (e.u.)		
**59 Vitaceae**										
Vitis vinifera L.	Enba,	Vite	Antiseptic	Tonic,	Fresh leaves	Leaves	Ground and	Locally	Both red or	7,12,15,16,17,20,23,
	Dalia			astringent			applied on	applied	white wine has	24,25,26,27,28,29,39
				Diuretic			wounds		many properties	41,47,48
									acknowledged	
			Epistaxis	Rheumatism,	Leaves	Skin of	Decoction		by the	
			Uterine hemorrhage	arthritis	Leaves	pressed	Decoction		official	
				sciatic nerve pains		grapes			medicine	
			Constipation		Leaves		To be eaten			
			Ocular diseases	As eye salve	Green	Sap	Sap as			
				In regulating	branches	Leaves	eye salve	Decoction		
				blood capillary				(e.u.)		
				vessel circulation						
				Hemorrhoids						
				Veins ulcer						
				Chilblains						
				Hemostatic		Dried		Powdered and		
				cicatrizant		leaves		locally applied		
				Herpes zoster		"Sapa" (=boiled must)		Sapa mixed with		
				(use known only				warmed and "scetti" (=a sort		
				in Sardinia region)				of wheat typical		
								meal of Sardinia)		
60 Zygophyllaceae										
Peganum harmala L.	Harmel	Armel	Rheumatism	Anti- Parkinson	Fresh whole	Seeds	Decoction as	Not specified		16,17,18,26,28,29
			of lower limbs		plant		footbath			
					also					
					seeds or root		Powder			
					ground to		boiled in			
					powder		olive oil			
							to massage			
							or to rub,			
			Rheumatism				alternatively		Side effects:	
			of lower		Seeds				hallucination	
			limbs		ground to					
					powder					
			Anuria and		Seeds ground		powder mixed with honey			
			dysuria		to powder		and taken per os			
			Backpain		Seeds		Slightly			
			(lumbago)				roasted and			
							ground to			
							a powder			
							added to			
							honey			
							and taken			
							per os			
			Hypotensive		Seeds					
			Hypoglycemic		Seeds					
			Purulent		Whole plant		Locally			
			conjunctivitis		dried and		applied			
			Blepharitis		ground to a					
			Eczema		powder					
			Antipyretic		Leaves		Decoction			
			cold							
			Inflammation of				Decoction			
			oral hollow				as gargling			

- e.u. : external use
- The common uses are pointed in bold
- The boxes rimed in bold point the very interesting uses
